# Temporal Trends of Racial and Socioeconomic Disparities in Population Exposures to Upstream Oil and Gas Development in California

**DOI:** 10.1029/2022GH000690

**Published:** 2023-03-23

**Authors:** David J. X. González, Claire M. Morton, Lee Ann L. Hill, Drew R. Michanowicz, Robert J. Rossi, Seth B. C. Shonkoff, Joan A. Casey, Rachel Morello‐Frosch

**Affiliations:** ^1^ Division of Environmental Health Sciences School of Public Health University of California, Berkeley Berkeley CA USA; ^2^ Department of Environmental Science, Policy, and Management University of California, Berkeley Berkeley CA USA; ^3^ Mathematical and Computational Science Program Stanford University Stanford CA USA; ^4^ PSE Healthy Energy Oakland CA USA; ^5^ Lawrence Berkeley National Laboratory Energy Technologies Area Berkeley CA USA; ^6^ Department of Environmental Health Sciences Columbia University New York NY USA; ^7^ Department of Environmental and Occupational Health Sciences University of Washington Seattle WA USA

## Abstract

People living near oil and gas development are exposed to multiple environmental stressors that pose health risks. Some studies suggest these risks are higher for racially and socioeconomically marginalized people, which may be partly attributable to disparities in exposures. We examined whether racially and socioeconomically marginalized people in California are disproportionately exposed to oil and gas wells and associated hazards. We longitudinally assessed exposure to wells during three time periods (2005–2009, 2010–2014, and 2015–2019) using sociodemographic data at the census block group‐level. For each block group and time period, we assessed exposure to new, active, retired, and plugged wells, and cumulative production volume. We calculated risk ratios to determine whether marginalized people disproportionately resided near wells (within 1 km). Averaged across the three time periods, we estimated that 1.1 million Californians (3.0%) lived within 1 km of active wells. Nearly 9 million Californians (22.9%) lived within 1 km of plugged wells. The proportion of Black residents near active wells was 42%–49% higher than the proportion of Black residents across California, and the proportion of Hispanic residents near active wells was 4%–13% higher than their statewide proportion. Disparities were greatest in areas with the highest oil and gas production, where the proportion of Black residents was 105%–139% higher than statewide. Socioeconomically marginalized residents also had disproportionately high exposure to wells. Though oil and gas production has declined in California, marginalized communities persistently had disproportionately high exposure to wells, potentially contributing to health disparities.

## Key Points

- Black, Hispanic, and socioeconomically marginalized people persistently had disproportionately high exposure to oil and gas development
- The widest observed disparities were for Black people residing in neighborhoods with the most intensive oil and gas production
- Disparities in exposure to oil and gas development may contribute to previously reported health disparities

## 1 Introduction

Oil and gas development near residences increases risks of exposure to water pollution, air pollution, noise, and other stressors that may adversely affect health and socioeconomic well‐being (Adgate et al., 2014; Allshouse et al., 2019; DiGiulio & Shonkoff, 2021; Garcia‐Gonzales, Shamasunder, & Jerrett, 2019; Garcia‐Gonzales, Shonkoff, et al., 2019; Gonzalez, Francis, et al., 2022; Hays et al., 2017; McKenzie et al., 2018; Shonkoff, Hill, & Domen, 2021; Shonkoff, Jordan, et al., 2015; Shonkoff, Maddalena, et al., 2015; Williams et al., 2021). Studies from California have reported that residing near new and active oil and gas wells is associated with higher risk of adverse reproductive and respiratory health outcomes (Gonzalez et al., 2020; Johnston et al., 2021; Shamasunder et al., 2018; Tran et al., 2020, 2021). Indeed, the California Oil and Gas Public Health Rulemaking Scientific Advisory Panel applied the Bradford Hill Criteria for Causation and concluded with a high degree of certainty that there was a causal association between living near oil and gas development and adverse respiratory and perinatal outcomes (Hill, 1965; Lucas & McMichael, 2005; Shonkoff, Morello‐Frosch, et al., 2021). Plugged and abandoned wells may also be harmful to health, due to emissions of non‐methane volatile organic compounds (Solis, 2022; Williams et al., 2021). Several studies have reported worse health outcomes among racially and socioeconomically marginalized people living near oil and gas development, and, in several regions in North America, marginalized communities have disproportionately high exposure to wells and associated infrastructure (Caron‐Beaudoin et al., 2018, 2019, 2022; Casey et al., 2019; Cushing et al., 2020; Gonzalez et al., 2020; Gonzalez, Nardone, et al., 2022; Kroepsch et al., 2019; Proville et al., 2022; Tran et al., 2020, 2021).

Observed health disparities may be attributable to disproportionately high exposure to chemical contaminants and other stressors from upstream oil and gas development, defined as activities that include preproduction (drilling new wells or re‐working and maintaining existing wells), production (extraction of oil and gas from active wells, oil‐water separation, hydrocarbon processing, etc.), and postproduction (retiring and plugging wells). Each stage of production is associated with environmental stressors that may plausibly contribute to adverse health outcomes. These stressors include emissions of air pollutants, groundwater contamination, persistent noise and odors, seismic activity, and social disruption (Adgate et al., 2014; Deziel, Clark, et al., 2022; Garcia‐Gonzales, Shonkoff, et al., 2019). Assessing individual‐ or area‐level exposure to wells based on proximity provides a holistic indicator for the aggregate hazards associated with oil and gas development (Deziel, Clark, et al., 2022).

Several studies have found that racially and socioeconomically marginalized populations have disproportionately high exposure to oil and gas wells in several parts of the United States (U.S.). Johnston et al. (2016) found that, in the Eagle Ford region in southern Texas, the proportion of people of color living near oil and gas disposal wells was significantly higher than the proportion of non‐Hispanic white people. A follow‐up study (Johnston et al., 2020) found that Hispanic residents had disproportionately high exposure to natural gas flaring. An analysis of wastewater injection wells in Ohio found that census block‐group level median household income was inversely associated with the presence of wells (Silva et al., 2018). Similarly, cross‐sectional analyses by the Natural Resources Defense Council and FracTracker Alliance report that racially and socioeconomically marginalized communities have disproportionately high exposure to oil and gas wells in Los Angeles and Kern Counties, California (Ferrar, 2020; Srebotnjak & Rotkin‐Ellman, 2014). Disparities have also been observed in Canada, with higher exposures to VOCs and trace metals among Indigenous women living in British Columbia compared to non‐Indigenous pregnant women (Caron‐Beaudoin et al., 2018, 2019, 2022). Exposure disparities have been documented dating back to the early twentieth century, with evidence that these disparities are attributable, at least in part, to structural racist policies such as redlining and housing segregation (Cumming, 2018; Gonzalez, Nardone, et al., 2022; Quam‐Wickham, 1998).

Despite widespread exposure to upstream oil and gas development, the extent of oil and gas‐related exposure disparities among racially and socioeconomically marginalized populations in California remains poorly characterized (Czolowski et al., 2017; Michanowicz et al., 2019). To our knowledge, these disparities have only been examined cross‐sectionally (i.e., at one point in time) and without accounting for the life cycle of oil and gas wells (from preproduction to active production to retirement).

In this study, we longitudinally examined sociodemographic characteristics of communities near upstream oil and gas wells across major stages of development in California. We had two overarching aims. First, we aimed to determine whether racially or socioeconomically marginalized populations in California had disproportionately high exposure to new, active, retired, and plugged wells. Among exposed populations, we also investigated the characteristics of communities with more intensive exposures as indicated by greater cumulative production volume (Gonzalez, Francis, et al., 2022). Second, we aimed to examine whether observed disparities were persistent throughout the study period beginning with the first set of data collected by the American Community Survey (ACS) in 2005 through 2019. Among communities that were exposed to any wells throughout the study period, we compared sociodemographic characteristics in communities where the intensity of well drilling and the volume of production increased versus places where these activities decreased. In additional sets of analyses, we examined differences among the three counties with the greatest number of people living within 1 km of wells: Los Angeles, Orange, and Kern. Collectively, over 90% of Californians residing near new, active, and retired wells during the study period lived in these three counties. Findings from this study may help affected residents, researchers, and policymakers to better understand environmental justice‐related concerns in the context of oil and gas development across California.

## 2 Methods

### 2.1 Study Design

To conduct our longitudinal assessment of disparities of exposure to upstream oil and gas development activities, we leveraged time‐series data on sociodemographic characteristics and upstream oil and gas development from 2005 to 2019. We divided our study period into three time periods to align with data available from the ACS: 2005–2009, 2010–2014, and 2015–2019. Estimates based on the ongoing ACS are available for single years and for multiple years, with aggregation up to 5 years. Data in the 5‐year ACS estimates are representative of the period of data collection and are considered more reliable for areas with small populations and for relatively small demographic groups (U.S. Census Bureau, 2022). For purposes of the current study, we began the study period in 2005 because this was the first year for which 5‐year (2005–2009) ACS data are available. The geographic units of observation for these analyses were census block groups, which are contiguous areas nested within census tracts that typically contain 600 to 3,000 residents (U.S. Census Bureau, 2022). To investigate temporal variation in oil and gas activities, we examined trends in the drilling of new wells, production of oil and gas at active wells, and retirement of wells post‐production. We also investigated proximity to wells that were plugged and abandoned prior to the study period.

### 2.2 Study Population

The study population comprised people who resided in California between 2005 and 2019. We used census block group‐level sociodemographic data from the 5‐year averaged ACS for three periods: 2005–2009, 2010–2014, and 2015–2019. With the added temporal component of these analyses, our unit of analysis was the block group‐period. We used data from block group‐periods when comparing aggregated state‐ and county‐level sociodemographic data.

We were specifically interested in estimating exposure among residents with the following racial/ethnic identities: Hispanic/Latinx, non‐Hispanic Asian, non‐Hispanic Black, non‐Hispanic white, American Indian or Alaska Natives, and those belonging to other marginalized racial/ethnic groups. For purposes of this study, we consider Hispanic/Latinx, non‐Hispanic Asian, non‐Hispanic Black, and American Indian and Alaska Natives as racially marginalized. Throughout the manuscript, we use the gender‐inclusive term Hispanic and Latinx. We also assessed exposure to oil and gas development activities on land managed by federally‐ or state‐recognized tribes. For this set of analyses, we used the American Indian/Alaska Native/Native Hawaiian Area National Shapefile provided by the U.S. Census Bureau, which includes the geographic extent of federally‐ and state‐recognized American Indian reservations as well as lands held in trust (U.S. Census Bureau, 2018).

We recognize that there is broad heterogeneity among people within each racial/ethnic group representing diverse histories and experiences of marginalization. However, disaggregated data on race/ethnicity and tribal affiliation were not available from ACS. In California, there are 109 federally recognized Indian tribes and 45 tribal communities that were formerly recognized or that have never been recognized by the federal government (California Tribal Court‐State Court Forum, n.d.). Tribal communities are located throughout the state, including in approximately 100 reservations or Rancherías as well as in urban settings. Los Angeles and San Francisco have some of the largest urban Indigenous populations in the U.S., including approximately 70,000 people with tribal origins in other states, many of whom are descendants of people who were forcefully relocated due to federal policy (California Tribal Court‐State Court Forum, n.d.). Among the Hispanic and Latinx population of California, in 2010, the majority (81.5%) were of Mexican descent, 4.1% were of Salvadoran descent, 2.4% were of Guatemalan descent, and 12.0% had familial origins in other countries (Ennis et al., 2011). Among the state's Asian population in 2010, 26.6% were of Filipino descent, 26.1% were of Chinese descent, 11.7% were of Vietnamese descent, 10.6% were of Asian Indian descent, 9.1% were of Korean descent, and 7.7% were of Japanese descent (Hoeffel et al., 2012). Additionally, several racially marginalized groups are subsumed under the non‐Hispanic white classification, such as many people of North African or Middle Eastern descent.

Finally, we also assessed exposure among residents who were socioeconomically marginalized. Specifically, we examined the following five indicators: proportion of residents with income below 200% of the federal poverty level, proportion of adults aged 25 and older with fewer than 12 years of education, proportion of renters, linguistically isolated people (i.e., adults 18 years or older who do not speak English “very well”), and proportion of eligible voters who did not participate averaged across the 2012 and 2016 elections. The U.S. government established their definition of the poverty level in the 1960s and has provided adjustments based on the initial definition. However, to meet basic needs, households require at least two times the federal level, which is why we based our definition on 200% of the federal poverty level (Su et al., 2009). Voter turnout data from the first study period (2005–2009) were unavailable and consequently we omitted voter turnout from our analyses of that time period.

### 2.3 Exposure Assessment

We obtained data on oil and gas wells from the California Geologic Energy Management Division (CalGEM), the state agency with regulatory authority over well permitting and many other aspects of oil and gas development in the state, as well as Enverus, a private data aggregation service. We assembled a data set that, for each well, included the geographic coordinates, spud date (the initiation of drilling and beginning of preproduction), completion date (end of preproduction), first and last dates of production, abandonment date, and well type. Additionally, we obtained data on the cumulative annual volume of oil and gas produced at each well, measured in barrels of oil equivalent (BOE).

**Figure 1:**
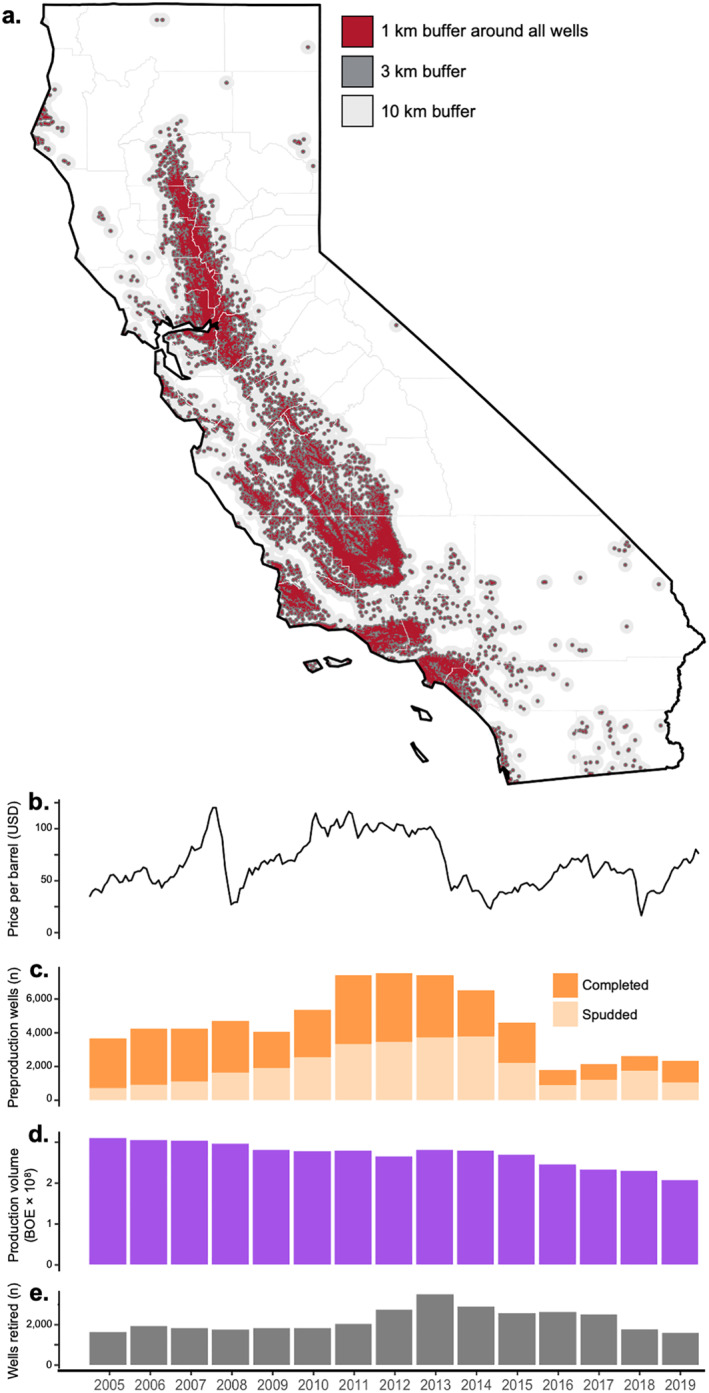
(a) Map of California showing 1, 3, and 10 km buffers around all wells in the preproduction, production, and postproduction stages during the study period, 2005–2019. (b) Midway‐Sunset first purchase price of a barrel of oil in U.S. dollars by month, an indicator for California oil prices. (c) Annual count of wells in preproduction in California, including wells spudded or completed. (d) Sum of annual oil and gas production volume in hundreds of millions of barrels of oil equivalent at all active wells. (e) Wells entering the postproduction stage (i.e., retired) by year, including plugged and long‐term idle wells (≥8 years without production).

We assessed exposure to all new, active, and retired wells across California (Figure 1). We defined new wells as those in the preproduction stage, between spudding (initiation of drilling) and completion (when the well is ready to start production). Active wells were defined as those with >0 BOE of cumulative oil or gas produced in any year of the study period. Finally, we defined retired wells as those that entered the postproduction phase during the study period. We defined the postproduction phase as starting on the reported date of abandonment or, where no abandonment date was reported, 8 years after the last production date in accordance with the state's definition of long‐term idle wells (California Code PRC § 3008). We also assessed exposure to all plugged wells in cross‐section for the 2015–2019 time period, as we expected any temporal variation in exposure to plugged wells during the study period to correlate with exposure to well retirements, which we assessed separately. Specifically, we defined plugged wells as those that were plugged and abandoned at any time, including wells retired before the study period began in 2005. In the plugged wells assessment, we included the 44.3% of plugged wells with no reported last production or abandonment dates, which we assumed were plugged prior to 2019. Notably, wells may have been in multiple production stages throughout the study period. In cases where a well was in two production stages during the same 5‐year time period (e.g., in preproduction in 2005 and production in 2006), we included it in both the new and active wells exposure assessments.

**Figure 2:**
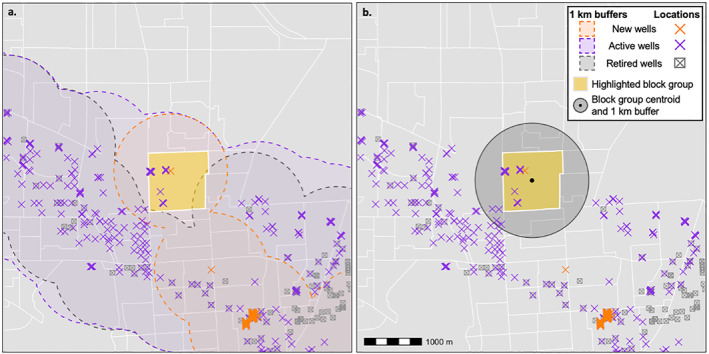
Illustration of the two exposure assessment protocols we used to estimate exposure to wells at the census block group‐level, with real data from Carson and Wilmington in Los Angeles County, predominantly Black and Latinx communities with intensive oil and gas production. Shown are a sample block group (highlighted in yellow) and all wells that were new, active, or retired during the study period, 2005–2019. (a) With the areal apportionment method our aim was to assess the proportion of residents who were exposed to wells in each stage of production, with the simplifying assumption that residents are evenly distributed throughout the block group. We assessed the proportion of each block group that intersected with 1 km buffers around new, active, or retired wells. In this example, all residents were exposed to new and active wells and approximately 10% were exposed to retired wells. (b) With the block group centroid method our aim was, among block groups with any exposure, to assess the intensity of the exposure, with the simplifying assumption that residents are in the center of the block group. We counted the number of wells in each development stage, and the sum of oil and gas production (barrels of oil equivalent), within 1 km of the block group centroid. In this example, neighborhood residents were exposed to 1 new well and 12 active wells (and the cumulative sum of oil and gas produced at those active wells).

For purposes of the current study, we defined exposed populations as those living within 1 km of oil and gas wells. In assessing exposure, we used a similar approach to proximity metrics commonly used in the environmental epidemiology literature (Deziel, Clark, et al., 2022). We assessed exposure for each census block group and time period (2005–2009, 2010–2014, and 2015–2019) with the aim first of estimating populations exposed to any wells and second, among exposed block groups, of estimating the intensity of production in BOE. First, we used areal apportionment to estimate populations that had any exposure to wells (Figure 2a). To do this, we determined the overlap between each block group and a 1 km buffer around wells in each production stage. We then determined the area of the block group that overlapped with the 1 km wells buffer and assigned exposure to residents in proportion to the area of overlap, with the simplifying assumption that residents were evenly distributed within the block group. For example, if a block group had 1,000 residents and 30% of the block group area intersected the well buffer, we assumed that 300 residents were exposed. We also apportioned residents within each sociodemographic group of interest in proportion to the percent overlap between the well buffer and block group. If, for example, 50% of residents in that same block group were Latinx, we would say that 150 of the exposed residents were Latinx. We completed this procedure for each block group‐period for wells in each stage of production. Second, among block groups with any exposure to wells, we ascertained the intensity of the exposure by taking the sum of wells or production volume within 1 km of the block group centroid (Figure 2b). Specifically, for each block group‐period, we counted the number of new, active, and retired wells (and plugged wells for 2015–2019). Additionally, for active wells, we assessed the cumulative sum of oil and gas production volume in BOE during that time‐period for all wells within 1 km of the block group centroid. As Deziel, Clark, et al. (2022) and Deziel, McKenzie, et al., 2022 have noted, proximity‐based exposure metrics have the advantages of being appropriate for retrospective analyses, easily scalable, and effective for capturing aggregate exposures to chemical, physical, and other stressors associated with oil and gas production, though the approach is constrained in not being able to identify specific etiological agents or risks beyond the buffer distance.

### 2.4 Statistical Analyses

We calculated summary statistics for exposure among each of the sociodemographic groups of interest identified above for each of the three time periods. We derived risk ratios to evaluate whether marginalized populations had disproportionately high exposure to wells. Specifically, to quantify the relative risk for each sociodemographic group of interest, we calculated a group risk ratio (RR):

$$\begin{array}{c} RR=\backslash frac\left\{\backslash frac\left\{\backslash sum\;Group\_\left\{exposed\right\}\right\}\left\{\backslash sum\;Group\_\left\{total\right\}\right\}\right\}\{\backslash frac\{\backslash sum\\ Population\_\left\{exposed\right\}\}\left\{\backslash sum\;Population\_\left\{total\right\}\right\}\}, \end{array}$$

or the ratio of the proportion of individuals in a specific demographic group who were exposed to the proportion of the total population who were exposed. A RR of greater than 1 indicates that the group was over‐represented among the exposed population. Conversely, groups that were under‐represented among the exposed population would have a RR of less than 1. Groups that were exposed in proportion that group's total population would have a RR equal to 1. For example, in 2005–2009, we estimated that 100,941 of the 2,172,247 (4.64%) non‐Hispanic Black residents of California resided within 1 km of an active well. We also estimated that 1,169,930 of all 36,308,527 (3.22%) California residents lived near active wells. Thus, the RR for this group was (100,941/2,172,247)/(1,169,930/36,308,527), or 1.44. (We could also have taken 4.64/3.22 to get to the same result). Since the RR was great than 1, we concluded that this demographic group had disproportionately high exposure in 2005–2009. Using this approach, we estimated the RR for each sociodemographic group of interest to determine whether they had disproportionately high or low exposure to oil and gas wells in each stage of production (new, active, retired, and plugged wells). We compared RRs through time among racial/ethnic groups and each of the socioeconomic indicators (people living in poverty, people with <12 years of educational attainment, renter‐occupied households, linguistically isolated, and non‐voters).

We also conducted these analyses at the county level for the three counties with the highest number of residents exposed to oil and gas development: Los Angeles, Orange, and Kern. For this set of analyses, we calculated RRs as described above, but with comparison to the county‐level demographic composition rather than the state‐level demographics.

In a secondary analysis, we analyzed the sociodemographic characteristics of block groups where there were changes in the count of new wells or sum of cumulative production volume between 2010–2014 and 2015–2019. We were interested in a panel data approach to investigating whether changes in intensity of oil and gas drilling and production were associated with the racial/ethnic composition of exposed block groups. Because the U.S. Census Bureau redefines block group boundaries at each decennial census, for this set of within‐block group analyses we used data for the latter two time periods. We then calculated the differences for both exposure metrics (new wells, cumulative production volume) between the two periods and categorized block groups by whether they had an increase, decrease, or no change for either metric. Among block groups within each category, we then summed the number of people in each racial/ethnic group.

### 2.5 Ethical Considerations

Because we used publicly available data for our analysis, our study does not fall within the definition of human subjects research.

## 3 Results

### 3.1 Demographics

In the first of the three time periods considered in this study (2005–2009), California had an estimated population of 36,308,527 residing in 22,133 census block groups (Table 1). The statewide population grew by 8.2% to 39,283,497 in 23,212 block groups by the third time period (2015–2019). The percentage of residents who were Hispanic or Latinx increased from 36.1% in 2005–2009 to 39.0% in 2015–2019. Similarly, the non‐Hispanic Asian population grew from 12.1% to 14.3% and the population that identified as two or more races increased from 2.1% to 3.0%. The proportion of the American Indian or Alaska Native population decreased slightly from 0.5% to 0.4%, the proportion of the non‐Hispanic Black population decreased from 6.0% to 5.5%, and the proportion of the non‐Hispanic white population decreased from 42.5% to 37.2%. The proportion of people living in poverty rose from 11.6% to 12.5%. With respect to educational attainment, the proportion of residents >25 years old who had not completed high school decreased from 19.5% in 2005–2009 to 16.7% in 2015–2019. The percent of renters increased from 42.1% to 45.2%. Between 20.0% and 21.9% of households were linguistically isolated during the study period. Across block groups, an average of 70.0 ± 10.9% of eligible voters participated in the 2012 and 2016 elections.

**Table 1:** Summary of Sociodemographic Characteristics of California Residents During Each of the Three Time Periods Considered in the Current Study

	Time period		
	2005–2009	2010–2014	2015–2019
California population, *n*	36,308,527	38,066,920	39,283,497
Census block groups, *n*	22,133	23,212	23,212
Race/ethnicity, *n* (%)			
Hispanic or Latinx	13,102,161 (36.1)	14,534,449 (38.2)	15,327,688 (39.0)
Non‐Hispanic nor Latinx			
American Indian or Alaska Native	164,754 (0.5)	145,736 (0.4)	140,831 (0.4)
Asian	4,410,395 (12.1)	5,062,736 (13.3)	5,610,931 (14.3)
Black	2,172,247 (6.0)	2,155,929 (5.7)	2,169,155 (5.5)
White	15,446,196 (42.5)	14,905,601 (39.2)	14,605,312 (37.2)
Other	126,774 (0.3)	81,869 (0.2)	100,119 (0.3)
Two or more races	762,608 (2.1)	1,044,136 (2.7)	1,188,673 (3.0)
Educational attainment, *n* (%)			
<12 years	4,537,564 (19.5)	4,602,986 (18.5)	4,418,675 (16.7)
12 years	5,078,536 (21.9)	5,153,257 (20.7)	5,423,462 (20.5)
>12 years	13,603,117 (58.6)	15,109,623 (60.8)	16,629,406 (62.8)
Living under 2x poverty line, *n* (%)	1,411,227 (11.6)	1,833,084 (14.5)	1,632,747 (12.5)
Renter‐occupied households, *n* (%)	5,125,759 (42.1)	5,708,355 (45.2)	5,889,686 (45.2)
Linguistically isolated adults, *n* (%)	5,893,258 (21.9)	6,171,673 (21.4)	6,038,794 (20.0)
Voter turnout, *n* (%)	—	13,360,940 (71.6)	13,360,940 (71.6)

*Note.* Percentages for educational attainment and living in poverty determined from reported education and poverty universes, respectively. Household‐level data for renters for the universe of California households. The percentage of linguistically isolated adults is calculated from the sum of total adult population of California during each time period. We reported average turnout and percent of voters among eligible residents using averaged data from the 2012 and 2016 elections; we applied these data in both the 2010–2014 and 2015–2019 periods.

### 3.2 Oil and Gas Wells

Across California, there were a total of 110,062 new, active, or retired wells between 2005 and 2019. There was substantial temporal variation in oil and gas operations. Some 30,713 wells were in preproduction during the study period, with drilling activity peaking in 2012 and decreasing through 2019 (Table S1 in Supporting Information S1). There were 83,583 active oil and gas wells in California during the study period, with production volume totaling 4.06 billion BOE. Among active wells, mean annual production was 24,754 ± 2,489,562 BOE (range: 0.1; 310,118,585). Statewide annual oil and gas production decreased by 33.5% throughout the study period, from 310 million BOE in 2005 to 206 million BOE in 2019 (Figure 1d). Some 31,199 wells (28.3%) were retired or long‐term idle (i.e., no production for at least 8 years) during the study period. Of these, 22,774 (73%) were plugged wells and 8,425 (27%) were long‐term idle. Well retirements increased from 1,639 in 2005 to 3,503 in 2013, then decreased to 1,604 in 2019. There were 126,560 total plugged wells in the data set, including wells retired before the study period. Of these, 100,697 (79.6%) had a reported spud or completion date, which ranged from March 1882 to December 2019. Abandonment dates ranged from February 1887 to June 2018. The duration of production for plugged wells was 10.9 ± 8.2 years, though duration data were missing for 70,700 plugged wells. A total of 56,039 (44.3%) of the plugged wells did not have reported last production or abandonment dates.

### 3.3 Proximity to and Risk Ratios for Exposure to Wells

We estimated that, from 2005 to 2009, 395,720 (1.1%) Californians lived within 1 km of new wells, 1,169,930 (3.2%) lived within 1 km of active wells, and 883,394 (2.4%) lived within 1 km of wells that were retired or long‐term idle (Table 2). The number and proportion of California residents living near wells decreased during the study period, with 170,679 (0.4%) living near new wells in 2015–2019, 1,109,293 (2.8%) living near active wells, and 588,647 (1.5%) near wells that were retired or long‐term idle. In 2015–2019, nearly 9.0 million (22.9%) California residents lived within 1 km of a well that had been plugged and abandoned. Note that some individuals were exposed to wells in multiple stages (e.g., both active and plugged wells).

**Table 2:** Estimates of the Number of Californians Residing Within 1 km of at Least One Well Stratified by Time Period

	Time period		
	2005–2009	2010–2014	2015–2019
New wells	395,720 (1.1)	214,497 (0.6)	170,679 (0.4)
Active wells	1,169,930 (3.2)	1,150,382 (3.0)	1,109,293 (2.8)
Retired wells	883,394 (2.4)	775,053 (2.0)	588,647 (1.5)
Plugged wells^a^	—	—	8,986,469 (22.9)

*Note.* We assessed exposure separately for new, active, retired, and (for 2015–2019) plugged wells. The values are reported as number (%) of residents exposed.

^a^ Includes wells plugged and abandoned at any time before 2019, including wells retired before the beginning of the study period in 2005.

#### 3.3.1 Proximity to New Wells

**Figure 3:**
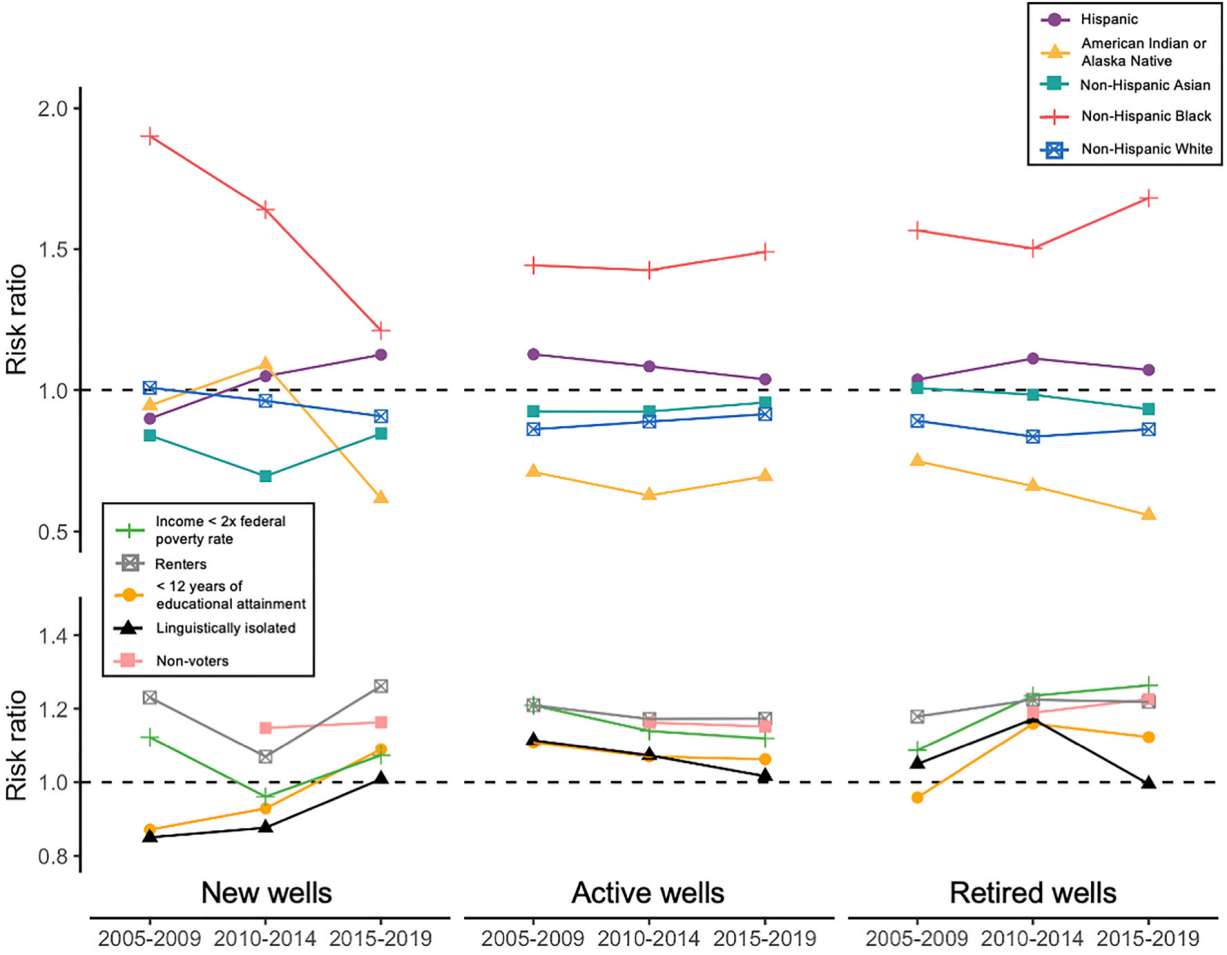
Estimated risk ratios for each racial/ethnic (top row) and socioeconomic group of interest (bottom). We estimated the proportion of each group with any exposure to wells in one of three stages: new wells (left column), active wells (center), and retired wells (right). For each group and well stage, we estimated risk ratios in the three time periods included in the study: 2005–2009, 2010–2014, and 2015–2019. A risk ratio >1 means the group has disproportionately high exposure compared to statewide representation of the group and a risk ratio <1 means the group has disproportionately low exposure, compared to the group's statewide representation.

Several marginalized groups had disproportionately high exposure to new wells during the study period. The total number of people exposed to new wells declined by 56.9% through the study period, which correlates with a decline in the number of wells that were drilled or spudding during the study period. The rate of decline was comparatively higher for non‐Hispanic white people (66.1%) and lower for Hispanic and Latinx people at 41.6% (Table S2 in Supporting Information S1). Non‐Hispanic Black people had the greatest disparities throughout the study period, though there was a steep decline, with RR ranging from 1.90 in 2005–2009 to 1.21 in 2015–2019 (Figure 3). American Indian and Alaska Native people had disproportionately high exposure in 2010–2014, with an of RR 1.09, declining to 0.62 in 2015–2019. Hispanic and Latinx people also had disproportionately high exposure starting in 2010, with an RR of 1.05 in 2010–2014 increasing to 1.13 in 2015–2019. Non‐Hispanic white people were not disproportionately exposed at any point and non‐Hispanic Asian people had disproportionately low exposure (RR < 1) in all three time periods. Low‐income people were more likely to live near new wells in 2005–2009 (RR = 1.12) and 2015–2019 (RR = 1.07), but not in 2010–2014 (RR = 0.96). Renters consistently had high exposure to new wells, with RRs ranging from 1.07 to 1.26. Non‐voters had disproportionately high exposure between 2010 and 2019, with an RR of 1.15.

#### 3.3.2 Proximity to Active Wells

We observed similar racial and socioeconomic disparities in residential proximity to active wells. Though there was not a substantial change in the total number of people living near active wells during the study period, the number of non‐Hispanic white people exposed decreased by 12.0% and exposed non‐Hispanic Black people decreased by 9.4%, while the numbers of exposed non‐Hispanic Asian people increased by 15.3% and multiracial people near active wells increased by 38.9% (Table S3 in Supporting Information S1). Despite these changes in absolute number of people exposed to active wells, non‐Hispanic Black people were still consistently disproportionately exposed, with an RR of 1.44 in 2005–2009 increasing slightly to 1.49 in 2015–2019. Latinx people also had disproportionately high exposure, with an RR of 1.13 in 2005–2009 declining to 1.04 in 2015–2019. Non‐Hispanic American Indian, Alaska Native, Asian, and white people had disproportionately low exposure to active wells throughout the study period (RR < 1). Socioeconomically marginalized people had disproportionately high exposure to active wells throughout the study period. The widest disparities were for renters with RRs between 1.17 and 1.21, non‐voters with RRs of 1.15 or 1.16, and low‐income people with RRs between 1.12 and 1.21.

#### 3.3.3 Proximity to Retired or Long‐Idle Wells

We found similar patterns of exposure disparities for populations near wells that were retired or long‐term idle. The number of people living near well retirements decreased by 33.4% during the study period, but non‐Hispanic white people had the fastest rate of decline at 43.7% (Table S4 in Supporting Information S1). Non‐Hispanic Black people were again consistently disproportionately exposed to retired wells, with an RR of 1.57 in 2005–2009 increasing to 1.68 in 2015–2019. Hispanic or Latinx people also had slight exposure disparities throughout the study period, with RR ranging from 1.04 to 1.07. Once again, American Indian, Alaska Native, non‐Hispanic Asian, and non‐Hispanic white people either were not disproportionately exposed or had comparatively low exposure. Low‐income people, renters, and non‐voters were consistently over‐represented among populations living near retired wells (RR > 1.1). Linguistically isolated adults had disproportionately high exposure from 2005 to 2014, and people who had not finished high school had higher exposure from 2010 to 2019.

#### 3.3.4 Proximity to Plugged and Abandoned Wells

Finally, there were also similar disparities in exposure to plugged and abandoned wells in 2015–2019, including those plugged before 2005. Non‐Hispanic Black people had the greatest exposure disparity (RR = 1.16), followed by Hispanic people (RR = 1.12), non‐Hispanic Asian people (RR = 1.04), and those categorized as non‐Hispanic other (RR = 1.04). We observed disproportionately low exposure among American Indian and Alaska Native people (RR = 0.68), non‐Hispanic white people (RR = 0.85), and non‐Hispanic multiracial people (RR = 0.88). By all indicators, socioeconomically marginalized people had disproportionately high exposure to plugged wells in 2015–2019. The widest disparities were for linguistically isolated adults, people who had not finished high school, and non‐voters.

### 3.4 Intensity of Exposure

Among exposed block groups within 1 km of active wells, the average production volume decreased by 28.7% from 670,918 BOE in 2005–2009 to 478,467 in 2015–2019 (Table S5 in Supporting Information S1). There was a wide range in block group‐level exposure to cumulative production volume, from a minimum of 10 BOE to a maximum of 14,100,859 BOE in 2005–2009. The number of block groups within 1 km of new wells decreased over the study period, with 1.3% exposed to new wells in 2005–2009 and 0.5% exposed in 2015–2019. The mean number of new wells in exposed block groups increased from 7.2 in 2005–2009 to a peak of 8.8 in 2010–2014, then decreased to 6.5 in 2015–2019. For active wells, 3.4% of block groups were exposed in 2005–2009 and 2.9% were exposed in 2015–2019. Exposed block groups had an average of 22.9 wells nearby in 2005–2009, increasing to an average of 24.3 in 2015–2019. Exposure to well retirements also decreased, from an average of 5.4 wells across 586 exposed block groups in 2005–2009 to an average of 4.1 wells across 367 block groups in 2015–2019.

**Figure 4:**
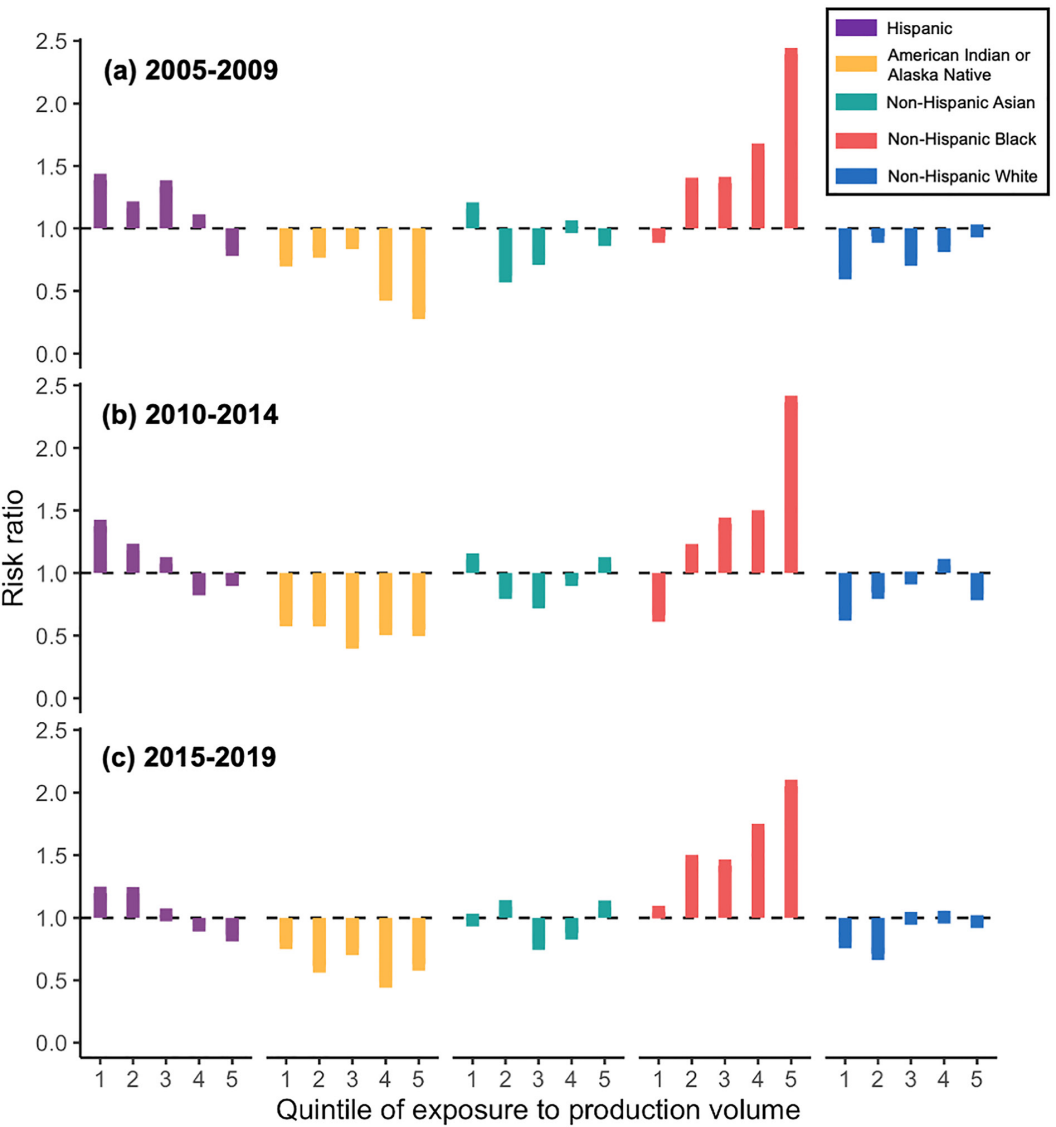
Estimated risk ratios for exposure to cumulative oil and gas production volume in barrels of oil equivalent stratified by quintile of exposure (from least exposure, 1, to highest exposure, 5), among block groups within 1 km of producing wells. For each racial/ethnic group, we estimated risk ratios by comparing the proportion of the group that was exposed relative to the proportion of the group statewide. A risk ratio >1 means the group has disproportionately high exposure compared to the statewide proportion and a risk ratio <1 means the group has disproportionately low exposure.

Among exposed block groups, as exposure to cumulative production volume increased, the percentage of the non‐Hispanic Black population also monotonically increased, with the largest exposure disparity in block groups with the most intensive production (Figure 4). In block groups in the lowest quintile of exposure to production volume, the RR for non‐Hispanic Black people ranged from 0.66 to 1.04 between the three time periods. Exposure disparities for non‐Hispanic Black people increased substantially among block groups in the higher exposure quintiles, ranging from 2.05 to 2.39 in the areas with the most intensive production. Hispanic and Latinx people only had disproportionately high exposure in block groups with relatively lower production intensity (i.e., in the first and second quintiles). Non‐Hispanic white people had disproportionately low exposure in areas with comparatively less production volume, but not in areas with high production volume. American Indian and Alaska Native people were not disproportionately exposed. These patterns were consistent across all three time periods. For linguistically isolated people and residents who had not finished high school, observed disparities were higher in areas with less production (Figure S1 in Supporting Information S1). There was not substantial variation in RRs for renters, low‐income people, and non‐voters.

We also considered exposure to different densities of new and retired wells. As we observed for production volume, non‐Hispanic Black people were more likely to live in areas with any drilling and, among those living near new wells, the exposure disparities were widest in areas with more intensive drilling (Figure S2 in Supporting Information S1). However, the pattern was different for well retirements. Non‐Hispanic Black people were more likely to live near retired wells, but, except for 2005–2009, the RRs were largest in areas with less intensive activity (Figure S3 in Supporting Information S1).

### 3.5 County‐Level Analyses

More than 90% of Californians residing near new, active, or retired wells during the study period lived in Los Angeles, Orange, or Kern Counties (Table S6 in Supporting Information S1). Los Angeles County had the most exposed residents in all well stages, with 123,314 residents residing near new wells, 725,503 near active wells, 431,051 near retired wells, and 4,663,082 near plugged wells. In Los Angeles County, from 2005 to 2019, non‐Hispanic Black people had disproportionately high exposure to oil and gas wells at each stage of production, similar to statewide results (Figure S6 in Supporting Information S1). Hispanic and Latinx people and non‐Hispanic white people also had disproportionately high exposure to new wells before 2015, patterns we did not see statewide. Renters were more likely to live near wells at all stages of production. Low‐income people had disproportionately high exposure to active and retired wells. People who were linguistically isolated or who had not finished high school had lower exposure to new wells. Los Angeles neighborhoods with more intensive oil and gas production also had higher proportions of non‐Hispanic Black people (Figure S7 in Supporting Information S1), but not socioeconomically marginalized people (Figure S8 in Supporting Information S1).

In Orange County, non‐Hispanic white people had disproportionately high exposure to wells in all development stages from 2005 to 2019 (Figure S9 in Supporting Information S1). American Indian and Alaska Native people living in Orange County had disproportionately high exposure new wells between 2005 and 2014 and to active and retired wells from 2005 to 2009. All other racial/ethnic groups had disproportionately low exposure. There were no substantial differences in these patterns when we examined areas with more intensive production for the racial/ethnic (Figure S10 in Supporting Information S1) and socioeconomically marginalized groups (Figure S11 in Supporting Information S1).

In Kern County, non‐Hispanic white people consistently had disproportionately high exposure to wells in all development stages (Figure S12 in Supporting Information S1). American Indian and Alaska Native people had higher exposure to wells in some stages. All other racial/ethnic groups in Kern County had disproportionately low exposure across well stages and throughout the study period. Disparities for non‐Hispanic white people were wider in areas with more intensive production, except in 2005–2009 (Figure S13 in Supporting Information S1). Socioeconomically marginalized people were typically less likely to live in neighborhoods near the most productive oil and gas wells (Figure S14 in Supporting Information S1).

### 3.6 Secondary Analyses

There was not substantial oil and gas development on federally‐ or state‐recognized tribal lands in California. A total 19 wells, all of which were idle or plugged, were located on the lands of the Paskenta Band of Nomlaki Indians (17 wells), the Morongo Band of Mission Indians (1 well), and the Soboba Band of Luiseño Indians (1 well).

In the within block group analyses, we did not observe substantial differences in the racial composition of block groups where the number of new wells changed between 2010–2014 and 2015–2019 (Figure S4 in Supporting Information S1). Non‐Hispanic Black people comprised a slightly higher proportion of people in block groups where the count of new wells decreased than in block groups where the count of new wells increased or stayed the same. Non‐Hispanic Asian people comprised a higher proportion of people in block groups where the number of new wells did not change. Similarly, we did not observe substantial racial/ethnic differences among block groups where cumulative production volume shifted (Figure S5 in Supporting Information S1). There was slightly higher representation of Latinx and non‐Hispanic Black people in block groups where cumulative production volume changed.

## 4 Discussion

In this longitudinal study, we found that racially and socioeconomically marginalized people had disproportionately high exposure to oil and gas development activities across California between 2005 and 2019. Specifically, the proportion of Black, Hispanic and Latinx, and low‐income people residing within 1 km of wells was substantially higher than their representation statewide. For non‐Hispanic Black people and people living in poverty, these disparities were persistent throughout the 15‐year study period. Individuals living in proximity to wells may be exposed to chemical hazards, noise, and other stressors associated with oil and gas developments (Allshouse et al., 2019; Elliott et al., 2017; Garcia‐Gonzales, Shonkoff, et al., 2019). Though oil and gas development activities—and the total number of people exposed to them—declined throughout the study period, the rate of decline was higher for non‐Hispanic white people and lower for Hispanic and Latinx people. We observed that while the disparities for non‐Hispanic Black people living near new wells declined through the study period, the disparities were consistent for active wells, indicating the persistence of these disparities once wells have been put into production. Among block groups exposed to active oil and gas wells, more intensive production was associated with higher proportion of Black residents, heightening potential health risks faced by these residents. However, we did not observe this pattern for socioeconomically marginalized people. The widest disparities, therefore, were for non‐Hispanic Black people in areas of Los Angeles County with the most intensive production, which had 2.4 times the proportion of Black residents compared to the statewide population.

Systemic factors affecting oil and gas‐related exposures for Indigenous people may be different than for other racially marginalized groups, as some tribes in some settings have permitted upstream oil and gas production on their tribally‐controlled land (Gilio‐Whitaker, 2019). We recognize that this may present a different dynamic than in areas where historically marginalized communities have lacked decision‐making power over local land uses and where tribal lands have been forcefully seized. In our analysis, we observed fewer than 20 wells on federally‐ or state‐recognized tribal lands in California. However, there may be additional wells located on lands held by tribes that are not federally‐ or state‐recognized and consequently not included in the data set we utilized. Importantly, Farrell et al. (2021) found that the estimated 606,604 square km of tribes' present‐day lands are less likely to overlie oil and gas reserves than the over 54 million square km of historical tribal lands, which the authors note has limited tribes' ability to benefit from resource extraction in ways that settlers have.

There were notable differences when we separated out the analysis by county in the three most exposed counties. The results for Los Angeles County mirrored the statewide results, likely because the county is home to most California residents who were exposed to new, active, retired, and plugged wells. Linguistically isolated residents and non‐voters had disproportionately low exposure to wells. The results for Orange and Kern Counties were substantially different from Los Angeles and state‐wide findings. There was disproportionately high exposure among non‐Hispanic white, and American Indian/Alaska Native residents for all well stages, and we did not observe disproportionately high exposure among socioeconomically marginalized residents of either Orange or Kern County. Notably, there are several predominantly Latinx communities in Kern County near active oil fields, some of which have successfully advocated for protections from oil and gas wells in their neighborhoods (Kane, 2020). Collectively, these results indicate that patterns of oil and gas development activities that lead to environmental inequities vary across California.

Land‐use decision‐making for extractive industries such as oil and gas involve many factors, including geological, economic, and policy considerations, particularly land‐use policies at municipal, county, and state‐levels (Kaufmann & Cleveland, 2001; Kroepsch et al., 2019). Prior studies indicate that racially and socioeconomically marginalized populations have less capacity to influence local land‐use policy and prohibit siting of industrial environmental hazards in their neighborhoods (Ash et al., 2013; Cushing et al., 2015). This is particularly important in communities facing cumulative environmental and social burdens, which may make residents more susceptible to exposure to stressors associated with oil and gas development (National Environmental Justice Advisory Council, 2004). Prior to the study period, historical structural factors appear to have influenced the siting of wells, including racist policies such as redlining that were promulgated by government agencies and private firms, discussed in greater depth below (Cumming, 2018; Gonzalez, Nardone, et al., 2022).

One in five Californians, or nearly 9 million people, lived within 1 km of a plugged and abandoned well in 2015–2019. The racial/ethnic and socioeconomic disparities in exposure to plugged and abandoned wells were similar to disparities observed for new and active wells. This may indicate that these disparities have been persistent before the study period and for wells that have long been abandoned, though demographic characteristics have likely changed in many areas with sustained oil and gas development. Hazards associated with plugged and abandoned wells are not well understood and likely vary by well type, underlying geology, well age, and other factors (Williams et al., 2021). However, due to data availability and scope, we did not assess these differences. The standards for well plugging and abandonment have varied through time, so wells retired before 1976, when more stringent standards were established, were held to less‐stringent requirements, and consequently may pose additional risks. In addition to wells that have been reported as plugged, another way to define postproduction wells include long‐term idle (8+ years), which may include unplugged “orphaned” wells (i.e., lacking a legally responsible owner) that must be plugged and abandoned at public expense. Finally, there are an unknown but potentially substantial number of legacy abandoned wells, or those that were drilled prior to consistent record‐keeping and which are not included in contemporary datasets, including wells that were never plugged. Methane emissions—often correlated with benzene, toluene, ethylene, and xylene, air toxics that have been associated with numerous adverse health outcomes—have been detected from abandoned wells (Gross et al., 2013; Lebel et al., 2020; McKenzie et al., 2017, 2018; Williams et al., 2021). Prior research has found that methane emissions from unplugged wells are worse than emissions from plugged wells and that gas wells have higher emissions than combined oil and gas wells (Lebel et al., 2020; Williams et al., 2021). Given the widespread potential exposures we observed, more study is needed to verify whether postproduction wells are indeed safe and to determine whether monitoring systems or policy interventions may help mitigate potential hazards.

There is a growing literature investigating associations between living near oil and gas development and adverse health outcomes, and documenting plausible etiologic pathways through which proximity to wells could confer risk (Deziel et al., 2020; Deziel, Clark, et al., 2022; Garcia‐Gonzales, Shonkoff, et al., 2019). The majority of epidemiological studies on this topic have reported significant associations (Deziel et al., 2020). Deziel et al. (2020) noted that among 12 studies that examined associations between proximity to oil and gas wells and risk of adverse birth outcomes, most observed positive relationships. Several perinatal studies employed a difference‐in‐differences study design, which helps control for unmeasured confounding, with each reporting positive associations between exposure to oil and gas development and adverse perinatal outcomes (Currie et al., 2017; Willis, Hill, Boslett, et al., 2021; Willis, Hill, Kile, et al., 2021). Deziel et al. (2020) found suggestive evidence of an association between proximity to oil and gas wells and risk of childhood cancer, though the studies in aggregate are as inconclusive. In a subsequent case‐control study, Clark et al. (2022) reported that exposure to unconventional oil and gas development was significantly associated with higher risk of childhood acute lymphoblastic leukemia.

Previously observed health risks may be attributable to multiple physical, chemical, and other stressors associated with oil and gas development (Deziel, Clark, et al., 2022). Contamination of groundwater from oil and gas‐related activities can result from spills, leaks from wells, and improper storage of wastewater, also referred to as produced water (Deziel, Clark, et al., 2022; DiGiulio et al., 2021). Noise pollution has been detected near oil and gas operations at levels that may cause sleep disturbance, annoyance, and increase the risk of cardiovascular disease (Allshouse et al., 2019; Blair et al., 2018; Hays et al., 2017). Numerous air pollutants are emitted at each stage of upstream oil and gas development. In a 2019 review, Garcia‐Gonzales, Shamasunder, and Jerrett (2019) and Garcia‐Gonzales, Shonkoff, et al. (2019) concluded that active oil and gas wells have the greatest potential to emit hazardous air pollutants, including several known carcinogens. Air monitoring in California found that drilling and operating oil and gas wells resulted in elevated levels of ambient air pollutants (including ozone and fine particulates) as far as 4 km downwind and at concentrations that could increase risk of adverse health outcomes (Gonzalez, Francis, et al., 2022; Gonzalez, Nardone, et al., 2022). Taken together, there is substantial evidence that exposure to oil and gas wells can contribute to a range of adverse health outcomes, mediated by air, water, and noise pollution, though additional studies that improve exposure assessment approaches can further elucidate specific etiologic pathways.

There is also prior evidence of disproportionately high exposure to oil and gas infrastructure and related health disparities among racially and socioeconomically marginalized people. A 2014 report found, among the subset of California communities already heavily burdened by pollution according to CalEnviroScreen 2.0, the majority (92%) of people living within 1 mile of wells were of Asian, Black, or Latinx descent (Srebotnjak & Rotkin‐Ellman, 2014). Casey et al. (2021) found that California residents of color and those with less political engagement are more likely to live near wells with higher methane emissions. Similar patterns have been observed in other states. Silva et al. (2018) found that injection wells were more likely to be sited in Ohio neighborhoods with lower income. In a study of exposure to natural gas flaring in Texas, Cushing et al. (2020) found risk of adverse birth outcomes was higher among Hispanic parents compared to non‐Hispanic white parents; the authors hypothesize that Hispanic pregnant people may be at higher risk due to disparities in preexisting chronic health conditions, co‐exposures to other pollutants, barriers to healthcare access, or other stressors associated with structural racism. Relatedly, two papers by Johnston et al. (2016, 2020) reported that Hispanic and Latinx people had disproportionately high exposure to wastewater disposal wells and natural gas flaring in Texas's Eagle Ford Shale. Clark et al. (2021) found that, though the number of oil and gas‐related complaints filed by residents in Pennsylvania did not vary by race/ethnicity, a determination that water supplies were impaired were less likely in areas with higher proportions of racially marginalized people. Proville et al. (2022) found that multiply marginalized populations intersected with more intensive oil and gas development in many areas of the U.S., including California's San Joaquin Valley. In a case‐control study, Gonzalez et al. (2020) found that exposure to oil and gas development was associated with higher risk of spontaneous preterm birth among birthing individuals in the San Joaquin Valley, California, and that the risk was confined to births to parents who were Hispanic, Black, or who had completed fewer than 12 years of education. Tran et al., in a set of retrospective cohort studies, found that proximity to active wells with higher oil and gas production volume (2020) and hydraulically fractured wells (2021) were associated with adverse fetal growth outcomes, including small‐for‐gestational age births, low birthweight births and lower term birth weight. In both studies, Tran et al. adjusted for known individual‐ and neighborhood‐level potential confounders identified a priori, and restricted their analyses to individuals within 10 km wells to reduce potential confounding from unobserved covariates. Also in both studies, Tran et al. observed strongest effects among rural residents. Notably, some studies that have reported racial and socioeconomic disparities have been cross‐sectional and may be subject to residual confounding.

Possible mechanisms leading to observed disparities in exposure to oil and gas development and associated health outcomes emerge from regulatory and land‐use decision‐making processes involving multiple stakeholders (Kroepsch et al., 2019). In the current study, we observed distributive injustice, with disproportionately high exposure among racial and socioeconomically marginalized people. Prior studies have examined the role of procedural injustice, including a racist policy known as redlining that, starting in the 1930s, designated neighborhoods as hazardous or high‐risk for investment or loans (Hillier, 2003, 2005; Swope et al., 2022). The real estate and banking industries, along with government agencies like the federal Home Owners Loan Corporation (HOLC), appraised neighborhood‐level investment risk and systematically assigned poorer grades to neighborhoods with Black, immigrant, and other marginalized groups (Hillier, 2003, 2005; Swope et al., 2022). In a case study of Los Angeles, Cumming (2018) describes how the racial composition of neighborhoods affected how HOLC staff graded neighborhoods, finding that in some predominantly white neighborhoods with oil wells, residents secured better grades by negotiating with HOLC staff. In other neighborhoods, the presence of both oil wells and people of color were cited as justification for assigning worse grades. Gonzalez, Nardone, et al. (2022) found that historical redlining was associated with the siting of oil and gas wells in 33 cities across the U.S., including Los Angeles. Neighborhoods with worse HOLC grades had significantly more wells, with the highest number and density of wells in historically redlined neighborhoods (Gonzalez, Nardone, et al., 2022). Historical redlining and other local, state, and federal policies that engendered and perpetuated racial segregation may partially account for the persistent racial disparities observed in the current study.

Our study had several limitations. Sociodemographic data are not publicly available at the individual level, so we were not able to identify individuals with intersecting marginalized identities. The U.S. Census Bureau provides data on some intersecting marginalized identities, and investigators should consider leveraging these data in future research. Because we assessed exposure at the area level, rather than the individual household, there is potential for exposure misclassification where, for example, residences are clustered on one side of a block group. This could be improved in future studies by determining where residences are located within census block groups, particularly for larger block groups (i.e., dasymetric mapping). The under‐reporting of legacy abandoned wells likely resulted in an underestimate of the populations exposed to these wells. Lebel et al. (2020) estimated that abandoned wells in California have been under‐counted by approximately 17%, based on historical maps from the U.S. Geological Survey. Williams et al. (2021) estimated that there are approximately 200,000 abandoned wells in California, which is roughly 74,000 or 58% more plugged and abandoned wells than were reported in the CalGEM data set we used in the current study. Due to insufficient plugging (or no plugging at all), these wells may emit air toxics that can be harmful to health, though to date there is only limited evidence of these potential hazards (Williams et al., 2021). More work is needed to identify locations of these wells and, importantly, whether insufficiently plugged wells indeed pose environmental health or safety risks for nearby residents. Additionally, examining exposure to other oil and gas infrastructure, such as pipelines and refineries, was outside our scope. However, due to potential risks associated with exposure to other infrastructure, more work assessing exposure to these potential hazards could help determine whether environmental inequities exist. There was also substantial missingness in last date of production or abandonment date. Some 44.3% of plugged wells had no reported date of last production or abandonment. Due to the long duration of oil and gas production in California, and the paucity of records from earlier periods, it is likely that many of these plugged wells were abandoned prior to 2005. Based on this, we do not expect there to be substantial misclassification of exposure to wells that were retired during the study period. In a secondary analysis, we assessed exposure to all wells labeled as plugged, and similarly observed that racially marginalized people were more likely to live near plugged wells regardless of whether an abandonment date was reported. Idle wells with a last date of production but no abandonment date may become active again, though the 8‐year period we used to define long‐idle abandoned wells accords with the state's definition of abandoned wells (California Code PRC § 3008).

We investigated whether racially and socioeconomically marginalized people in California were more likely to live in neighborhoods with oil and gas wells in all stages of production. Black, Latinx, and socioeconomically marginalized people had disproportionately high exposure to new, active, retired, and plugged oil and gas wells between 2005 and 2019. Black people not only had disproportionately high exposure to any wells; when they were exposed, they were more likely to be in areas with the most intensive production. Exposure to wells at each stage of development presents risks to health, and health disparities reported in prior studies may be partly attributable to disparities in exposure to wells. Across California, state and local policymakers have considered options to provide protections for frontline communities near oil and gas development facilities. Potential policy interventions aimed at mitigating risks associated with oil and gas development include on‐site engineering controls, setbacks or buffer distances between wells and sensitive receptors (e.g., residences, schools, medical facilities), and the elimination of oil and gas production (Deziel, McKenzie, et al., 2022). In September 2022, the California legislature passed a new statute that requires a 1 km setback between new wells and sensitive receptors, including residences (Senate Bill No. 1137, 2022). Jurisdictions such as Los Angeles and Culver City have banned new drilling, and Ventura County and the city of Arvin have instituted setback policies and restrictions on drilling and production (Kane, 2020). In forging potential interventions, policymakers should also address environmental health disparities. Further study could expand knowledge on hazards associated with living near oil and gas wells and mechanisms that have led to observed racial and socioeconomic health disparities.

## Conflict of Interest

The authors declare no conflicts of interest relevant to this study.

## Supporting information

Supporting Information S1

## Data Availability

Sociodemographic data used in the analyses presented here were obtained from the publicly available ACS conducted by the U.S. Census Bureau, specifically, 5‐year estimates for 2005–2009, 2010–2014, and 2015–2019. We obtained publicly available data on oil and gas development from the California Geologic Energy Management Division (CalGEM). Additional data came from the Enverus, a private oil and gas data aggregation service; academic researchers may request access to these data for research purposes. All analytic work and visualizations presented in this paper were done using R v. 4.2 software (R Core Team, 2022) as well as the “tidyverse” and “sf” packages (Pebesma, 2018; Wickham et al., 2019). The codebase and processed data set used in these analyses are licensed under MIT, and the GitHub repostory (v1.0.3) with these resources is availble on the FAIR‐compliant Zenodo platform at https://doi.org/10.5281/zenodo.7747408 (Gonzalez & Morton, 2023).
