# Supplementary material for: Temporal Trends of Racial and Socioeconomic Disparities in Population Exposures to Upstream Oil and Gas Development in California
Source: Geohealth. 2023 Mar 23;7(3):e2022GH000690. doi: 10.1029/2022GH000690 (PMC10035325; doi:10.1029/2022GH000690)
Supplement: Supplementary file 1 — Supporting Information S1 [file GH2-7-e2022GH000690-s001.pdf]

Supporting Information

Temporal trends of racial and socioeconomic disparities in population exposures to upstream oil and gas development in California

David J.X. González, Claire M. Morton, Lee Ann L. Hill, Drew R. Michanowicz, Robert J. Rossi, Seth B.C. Shonkoff, Joan A. Casey, and Rachel Morello-Frosch

Contents

Tables S1–S6
Figures S1– S14

Table S1. Summary statistics for wells that were new, active, or retired during the study period. We also considered wells that were plugged at any time prior to the end of the study period, including wells plugged before 2005. Note that some new wells were in preproduction in multiple time periods; additionally, some active wells were in continuous production during multiple time periods.

	Time Period			
	2005-2009	2010-2014	2015-2019	All
New wells, n	13,163	10,325	8,097	30,713
Active wells, n	64,823	68,430	63,497	85,583
Retired wells, n	9,028	13,052	11,119	33,199
Production volume, BOE*	1,495,061,879	1,382,923,470	1,183,983,473	4,061,968,822
Plugged wells†, n	—	—	126,560	—

* Total oil and gas production in BOE, or barrels of oil equivalent. † Includes wells plugged and abandoned at any time before 2019, including wells retired before the beginning of the study period in 2005.

Table S2. Estimated number and percentages of California residents exposed to <u>new wells</u> during each study period, stratified by racial/ethnic group.

	Time period		
	2005-2009	2010-2014	2015-2019
California population exposed, n	395,720	214,497	170,679
Race/ethnicity, n (%)			
Hispanic or Latinx	128,422 (32.5)	85,929 (40.1)	74,948 (43.9)
Non-Hispanic American Indian or Alaska Native	1,698 (0.4)	895 (0.4)	377 (0.2)
Non-Hispanic Asian	40,359 (10.2)	19,833 (9.2)	20,626 (12.1)
Non-Hispanic Black	45,002 (11.4)	19,923 (9.3)	11,411 (6.7)
Non-Hispanic white	169,751 (42.9)	80,799 (37.7)	57,587 (33.7)
Non-Hispanic other	1,164 (0.3)	471 (0.2)	642 (0.4)
Non-Hispanic two or more races	8,640 (2.2)	6,123 (2.9)	4,430 (2.6)

Sociodemographic were obtained data from the 5-year American Community Survey for the 2005–2009, 2010–2014, and 2015–2019.

Table S3. Estimated number and percentages of California residents exposed to active wells during each study period, stratified by racial/ethnic group.

	Time period		
	2005-2009	2010-2014	2015-2019
California population exposed, n	1,169,930	1,150,382	1,109,293
Race/ethnicity, n (%)			
Hispanic or Latinx	457,670 (39.1)	476,089 (41.4)	449,423 (40.5)
Non-Hispanic American Indian or Alaska Native	3,770 (0.3)	2,762 (0.2)	2,763 (0.2)
Non-Hispanic Asian	131,431 (11.2)	141,398 (12.3)	151,483 (13.7)
Non-Hispanic Black	100,941 (8.6)	92,802 (8.1)	91,304 (8.2)
Non-Hispanic white	428,752 (36.6)	400,141 (34.8)	377,349 (34.0)
Non-Hispanic other	4,188 (0.4)	2,784 (0.2)	3,250 (0.3)
Non-Hispanic two or more races	21,747 (1.9)	30,743 (2.7)	30,196 (2.7)

Sociodemographic were obtained data from the 5-year American Community Survey for the 2005–2009, 2010–2014, and 2015–2019.

Table S4. Estimated number and percentages of California residents exposed to well retirements during each study period, stratified by racial/ethnic group.

	Time period		
	2005-2009	2010-2014	2015-2019
California population exposed, n (%)	883,394	775,053	588,647
Race/ethnicity, n (%)			
Hispanic or Latinx	330,626 (37.4)	329,075 (42.5)	246,088 (41.8)
Non-Hispanic American Indian or Alaska Native	2,999 (0.3)	1,959 (0.3)	1,176 (0.2)
Non-Hispanic Asian	108,064 (12.2)	101,423 (13.1)	78,394 (13.3)
Non-Hispanic Black	82,768 (8.4)	65,955 (8.5)	54,643 (9.3)
Non-Hispanic white	334,972 (37.3)	253,531 (32.7)	188,484 (32.0)
Non-Hispanic other	2,987 (0.3)	1,969 (0.3)	1,862 (0.3)
Non-Hispanic two or more races	18,285 (2.1)	18,876 (2.4)	15,822 (2.7)

Sociodemographic were obtained data from the 5-year American Community Survey for the 2005–2009, 2010–2014, and 2015–2019.

Table S5. Distribution of exposure to wells and production volume within 1 km of census block groups centroids. Restricted to block groups in California with any exposure, stratified by time period and well production stage. Reported as median; mean ± SD (range), followed by the number and % of block groups that had any exposure.

	Time Period		
	2005–2009	2010–2014	2015–2019
New wells, n	2; 7.2 ± 19.8 (1; 120)	2; 8.8 ± 14.6 (1; 61)	2; 6.5 ± 14.6 (1; 122)
Block groups exposed (%)	285 (1.3)	137 (0.6)	112 (0.5)
Active wells, n	11; 22.9 ± 31.2 (1; 234)	11; 24.5 ± 35.8 (1; 218)	12; 24.3 ± 35.8 (1; 236)
Block groups exposed (%)	743 (3.4)	702 (3.0)	670 (2.9)
Well retirements, n	3; 5.4 ± 7.9 (1; 124)	2; 4.8 ± 6.9 (1; 82)	2; 4.1 ± 4.5 (1; 28)
Block groups exposed (%)	586 (2.6)	472 (2.0)	367 (1.6)
Cumulative production volume, BOE	150,661; 670,918 ± 1,413,897 (10; 14,100,859)	128,260; 548,760 ± 1,198,841 (60; 10,844,378)	112,378; 478,467 ± 1,018,029 (10; 8,426,452)

BOE, barrels of oil equivalent

Table S6. Estimated number of people living within 1 km of new, active, and retired wells during the most recent time period considered in the study (2015–2019), stratified by county.

County	New wells	Active wells	Retired wells	Plugged wells
Alameda	0	209	0	36,645
Alpine	0	0	0	0
Amador	0	0	0	74
Butte	0	66	6	3,797
Calaveras	0	0	0	0
Colusa	78	1,079	649	9,191
Contra Costa	2,049	11,564	638	224,016
Del Norte	0	0	0	236
El Dorado	0	0	0	0
Fresno	1,457	5,379	3,276	51,943
Glenn	42	1,264	973	15,958
Humboldt	0	685	307	7,500
Imperial	0	0	0	2,513
Inyo	0	0	0	0
Kern	17,873	125,016	49,533	529,154
Kings	1	1,048	379	10,643
Lake	0	0	0	33
Lassen	0	0	0	18
Los Angeles	123,314	725,503	432,051	4,663,082
Madera	0	41	20	7,027
Marin	0	0	0	190
Mariposa	0	0	0	0
Mendocino	0	0	0	874

Merced	0	0	33	12,795
Modoc	0	0	0	2
Mono	0	0	0	1
Monterey	47	67	76	24,167
Napa	0	0	0	148
Nevada	0	0	0	0
Orange	19,678	178,957	68,529	1,440,930
Placer	0	0	0	430
Plumas	0	0	0	0
Riverside	0	10	165	89,124
Sacramento	162	482	631	210,241
San Benito	0	7	706	7,276
San Bernardino	0	7,360	432	140,425
San Diego	0	0	0	82,229
San Francisco	0	0	0	0
San Joaquin	0	5,552	6,725	280,101
San Luis Obispo	199	329	249	37,706
San Mateo	0	95	78	18,687
Santa Barbara	1,490	13,084	3,713	197,088
Santa Clara	0	65	0	36,144
Santa Cruz	0	11	0	13,953
Shasta	0	0	0	1,367
Sierra	0	0	0	0
Siskiyou	0	0	0	9
Solano	30	3,828	4,201	53,058
Sonoma	0	0	0	13,429
Stanislaus	0	93	0	40,834
Sutter	0	1,361	671	9,454
Tehama	0	1,084	497	13,329
Trinity	0	0	0	0
Tulare	0	703	319	48,334
Tuolumne	0	0	0	0
Ventura	4,260	22,461	12,246	555,026
Yolo	0	1,849	1,539	94,857
Yuba	0	24	0	2,439

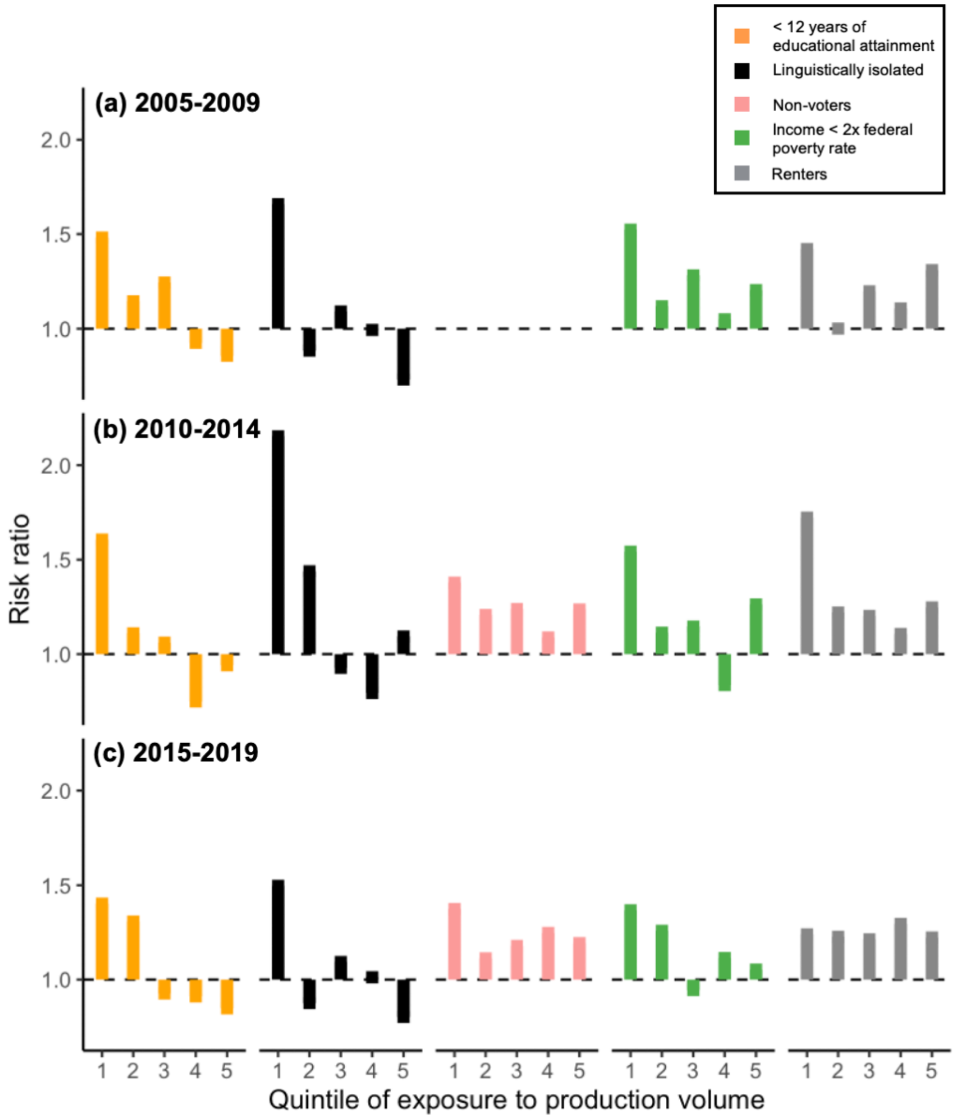

Figure S1. Among block groups within 1 km of producing wells, we estimated risk ratios for exposure to cumulative oil and gas production volume stratified by quintile of exposure (from least exposure, 1, to highest exposure, 5). For each indicator of socioeconomic marginalization, we estimated risk ratios by comparing the proportion of the group that was exposed relative to the proportion of the group statewide. A risk ratio > 1 means the group has disproportionately high exposure compared to the statewide proportion and a risk ratio < 1 means the group has disproportionately low exposure. Sociodemographic were obtained data from the 5-year American Community Survey for the 2005–2009, 2010–2014, and 2015–2019. For 2005–2009, the cutoffs between quantiles were: 22,500; 85,000; 266000; and 1,062,639 BOE. For 2010–2014, the cutoffs between quantiles were: 19,189; 62,000; 256,000; and 790,000; BOE. Finally, for 2015–2019, the cutoffs between quantiles were: 17,969; 51,440; 250,000; and 568,000 BOE. Compare to Figure 4.

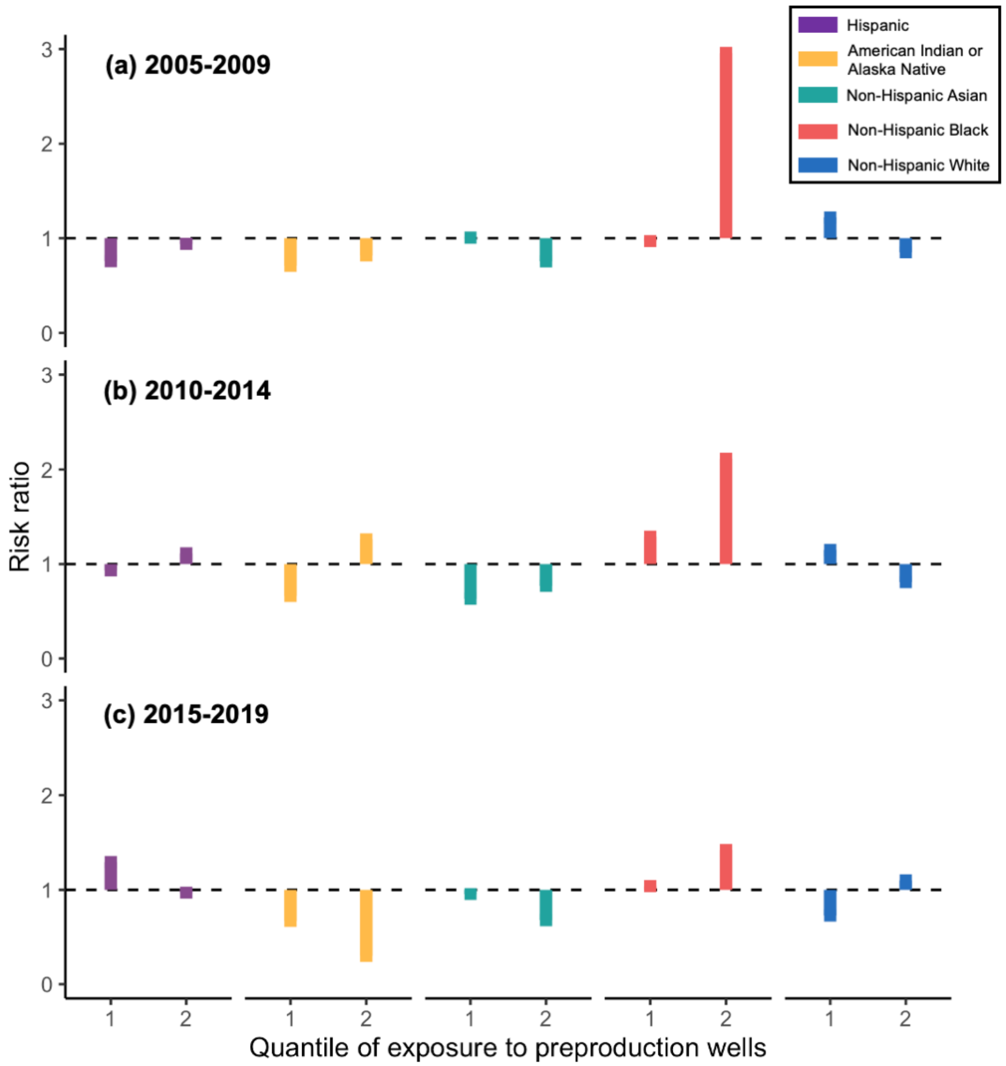

Figure S2. Among block groups within 1 km of new wells (i.e., in preproduction), we estimated risk ratios for exposure to new wells stratified by quantile of exposure (low exposure, 1, and high exposure, 2). For each racial/ethnic group, we estimated risk ratios by comparing the proportion of the group that was exposed relative to the proportion of the group statewide. A risk ratio > 1 means the group has disproportionately high exposure compared to the statewide proportion and a risk ratio < 1 means the group has disproportionately low exposure. For all time periods, the cutoff between quantiles was 2 wells. Compare to Figure 4.

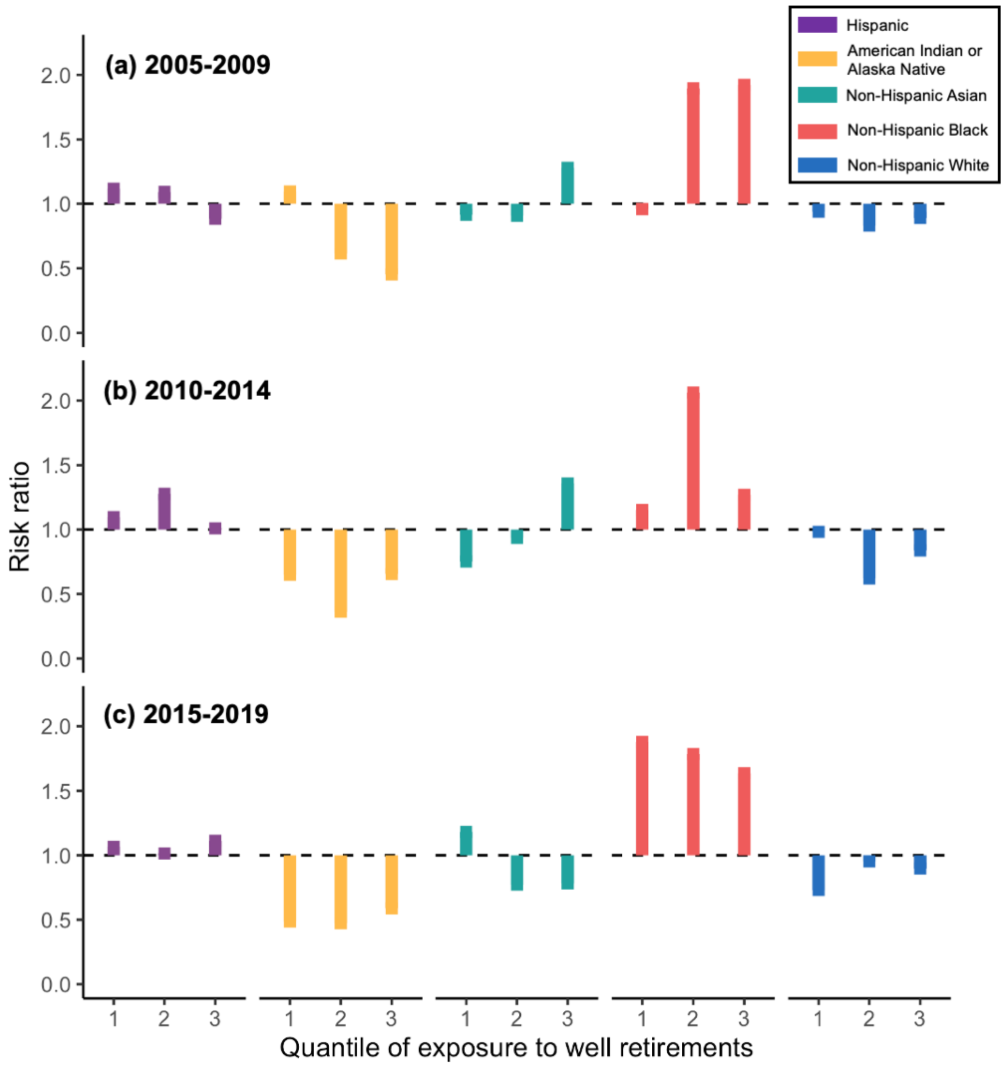

Figure S3. Among block groups within 1 km of retired wells (i.e., in postproduction), we estimated risk ratios for exposure to well retirements stratified by quantile of exposure (from low exposure, 1, to high exposure, 3). For each racial/ethnic group, we estimated risk ratios by comparing the proportion of the group that was exposed relative to the proportion of the group statewide. A risk ratio > 1 means the group has disproportionately high exposure compared to the statewide proportion and a risk ratio < 1 means the group has disproportionately low exposure. For 2005–2009, the cutoffs between quantiles were 2 wells and 5 wells. For 2010–2014, the cutoffs were 2 and 4 wells. Finally, for 2015–2019, the cutoffs between quantiles were again 2 and 4 wells. Compare to Figure 4.

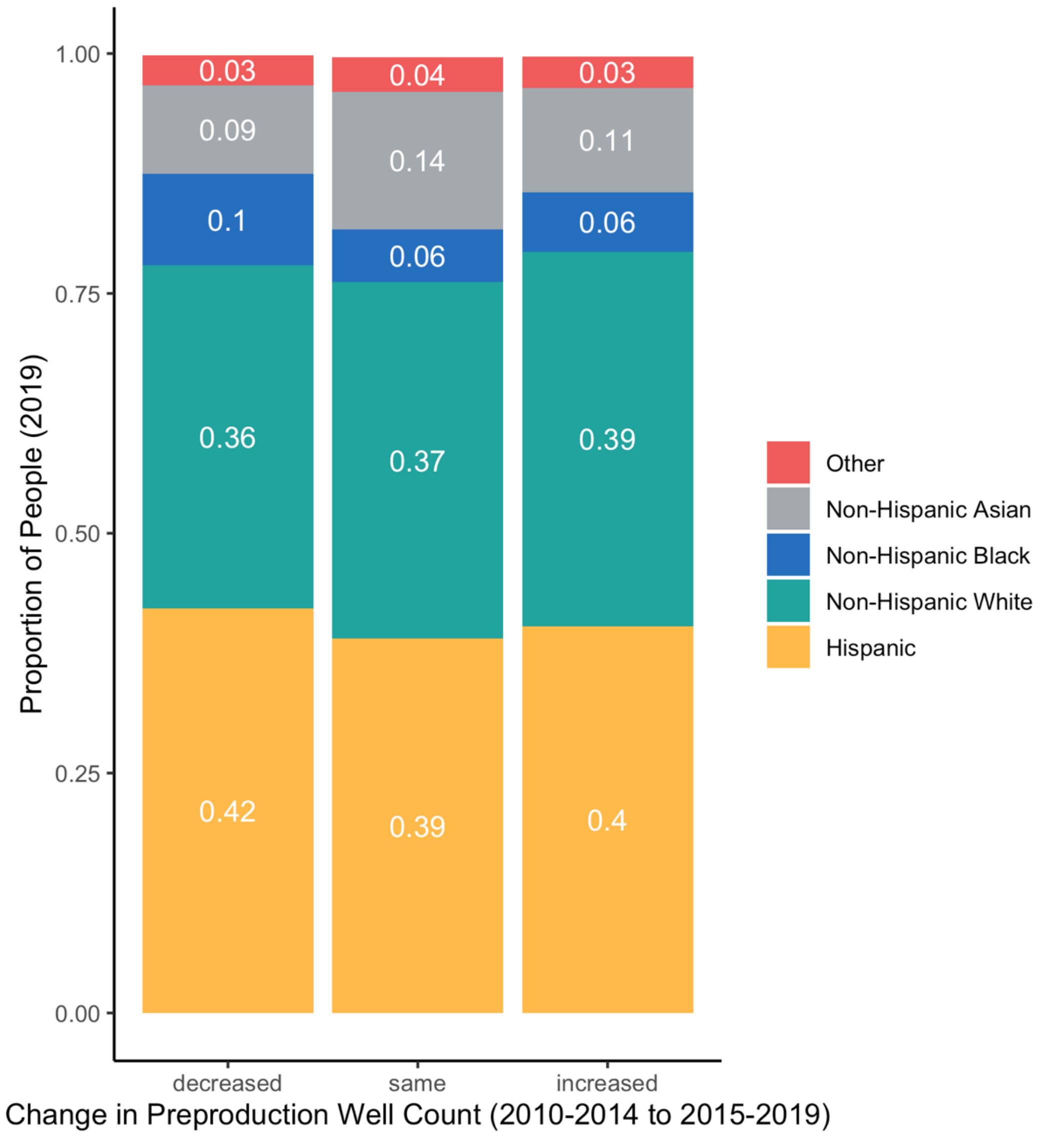

Figure S4. Results for within block group analyses for the racial/ethnic makeup of block groups where the count of new wells changed between 2010–2014 and 2015–2019. Restricted to block groups with any exposure to new wells.

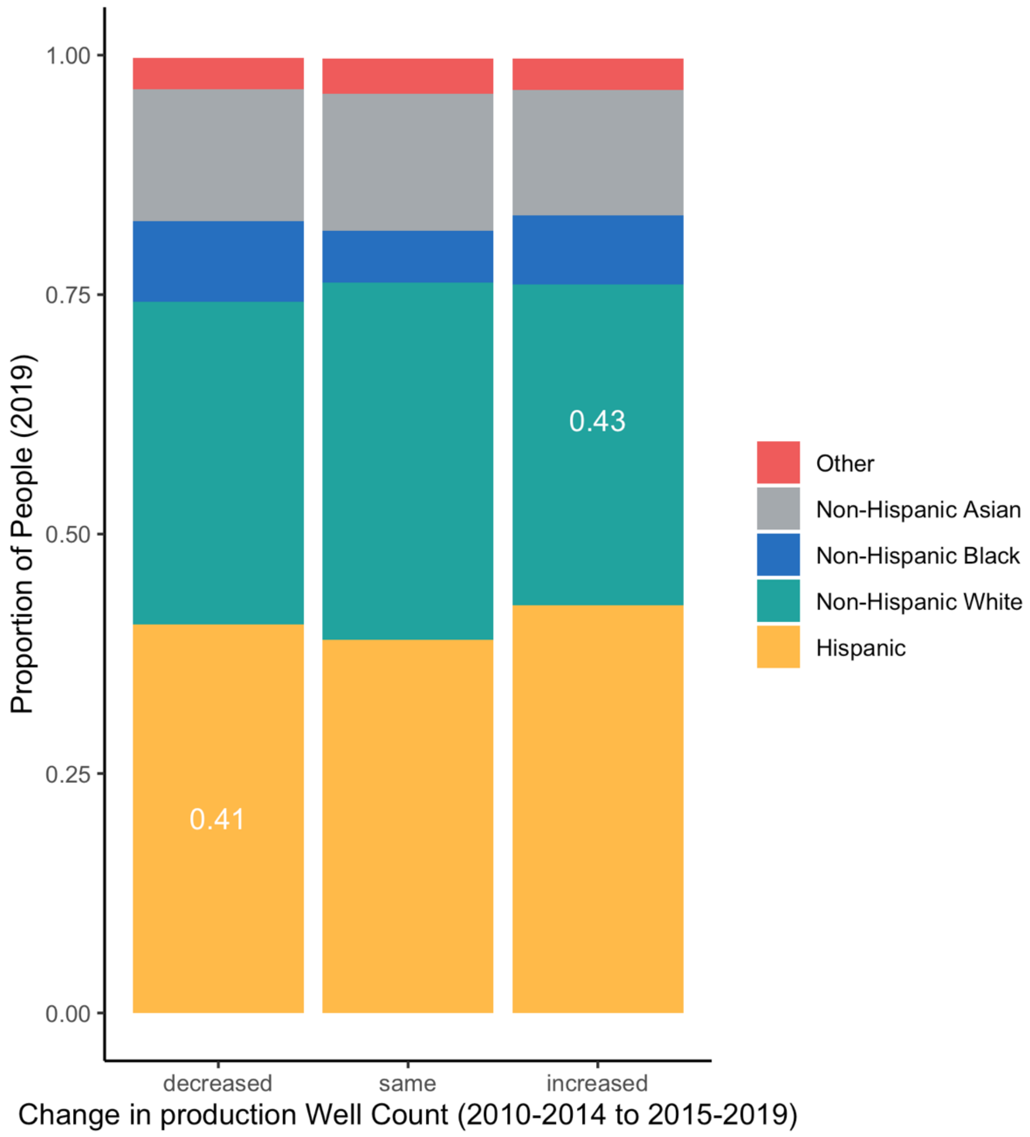

Figure S5. Results for within block group analyses for the racial/ethnic makeup of block groups where the sum of cumulative production volume changed between 2010–2014 and 2015–2019. Restricted to block groups with any exposure to active wells.

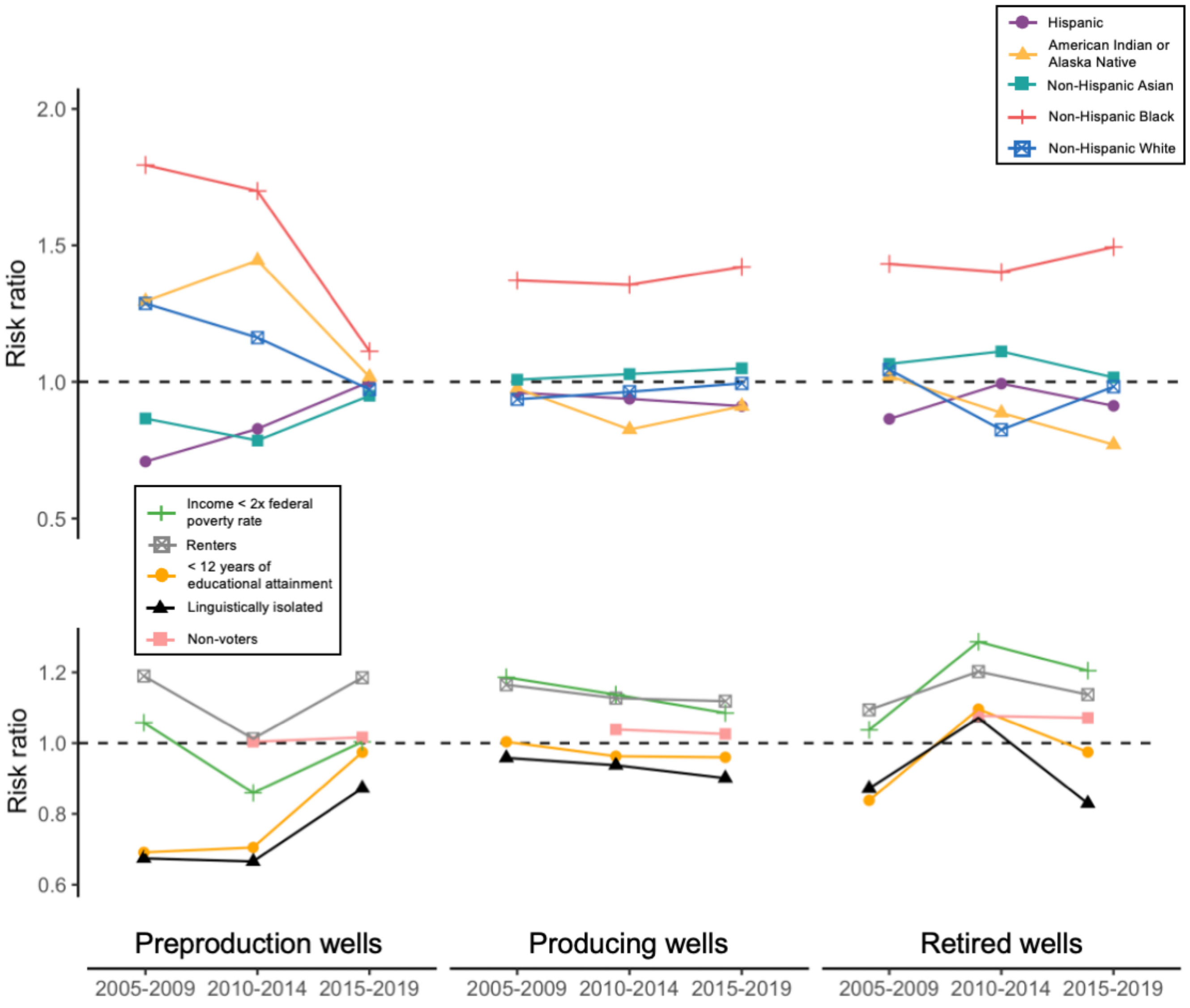

Figure S6. Estimated risk ratios for each racial/ethnic (top row) and socioeconomic group of interest (bottom). We estimated the proportion of each group in Los Angeles County with any exposure to wells in one of three stages: new (left column), active (center), and retired (right). For each group and well stage, we estimated risk ratios in the three time periods included in the study: 2005 to 2009, 2010 to 2014, and 2015 to 2019. A risk ratio > 1 means the group has disproportionately high exposure compared to the Los Angeles County representation of the group and a risk ratio < 1 means the group has disproportionately low exposure, compared to the group's representation in Los Angeles County. Compare to Figure 3.

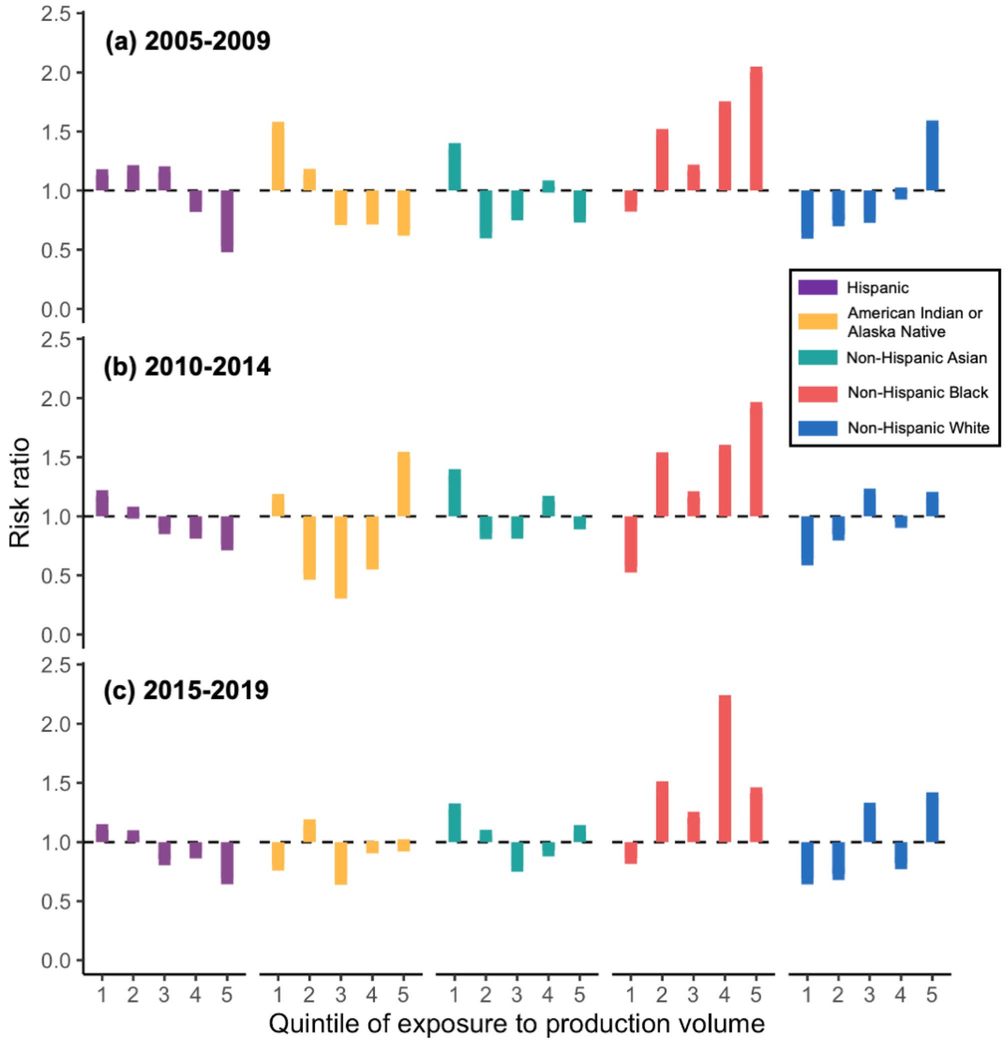

Figure S7. Among block groups in Los Angeles County within 1 km of active wells, we estimated risk ratios for exposure to cumulative oil and gas production volume stratified by quintile of exposure (from least exposure, 1, to highest exposure, 5). For each racial/ethnic group, we estimated risk ratios by comparing the proportion of the group that was exposed relative to the proportion of the group within the county. A risk ratio > 1 means the group has disproportionately high exposure compared to the county-wide proportion and a risk ratio < 1 means the group has disproportionately low exposure. Compare to Figure 4.

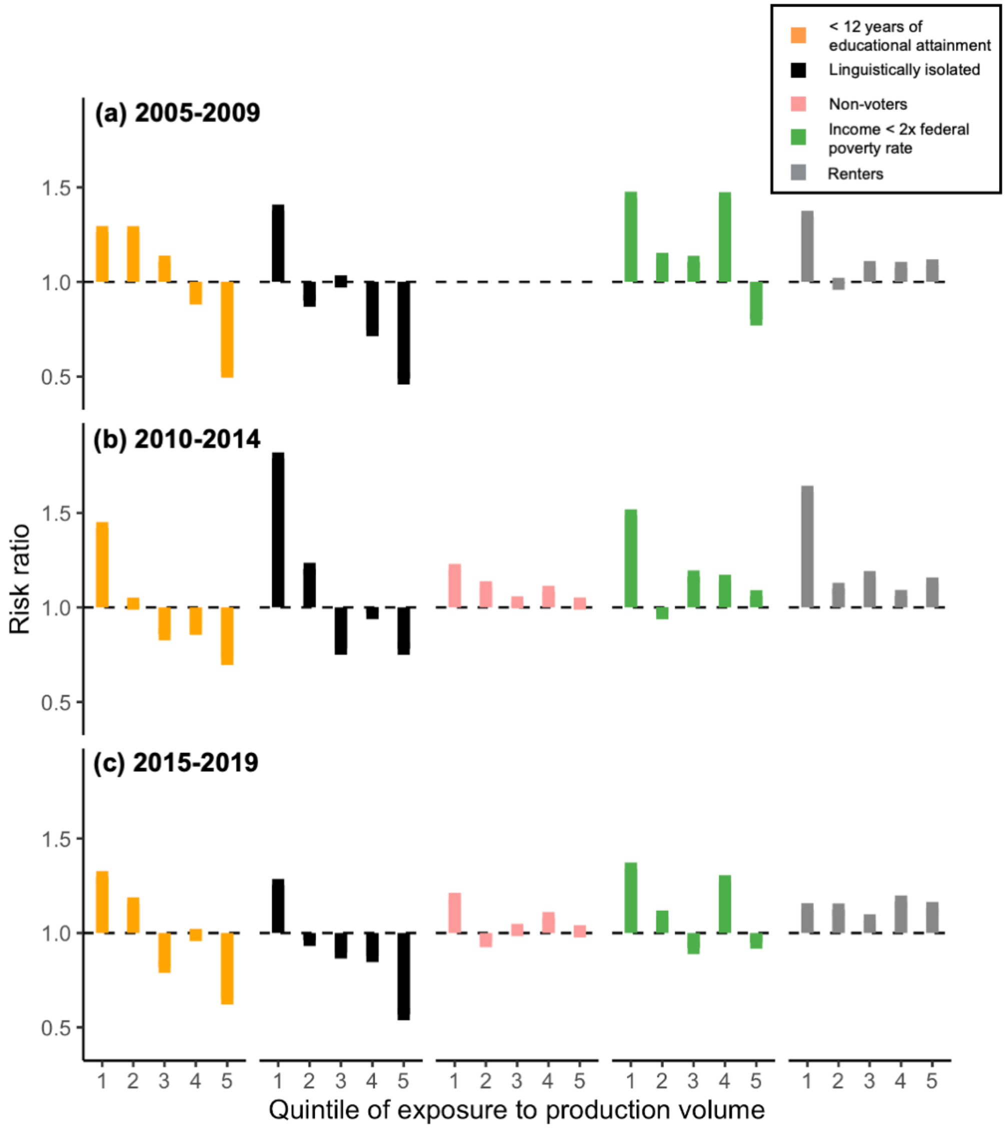

Figure S8. Among block groups in Los Angeles County within 1 km of active wells, we estimated risk ratios for exposure to cumulative oil and gas production volume stratified by quintile of exposure (from least exposure, 1, to highest exposure, 5). For each group identified by socioeconomic indicators, we estimated risk ratios by comparing the proportion of the group that was exposed relative to the proportion of the group within the county. A risk ratio > 1 means the group has disproportionately high exposure compared to the county-wide proportion and a risk ratio < 1 means the group has disproportionately low exposure. Compare to Figure S1.

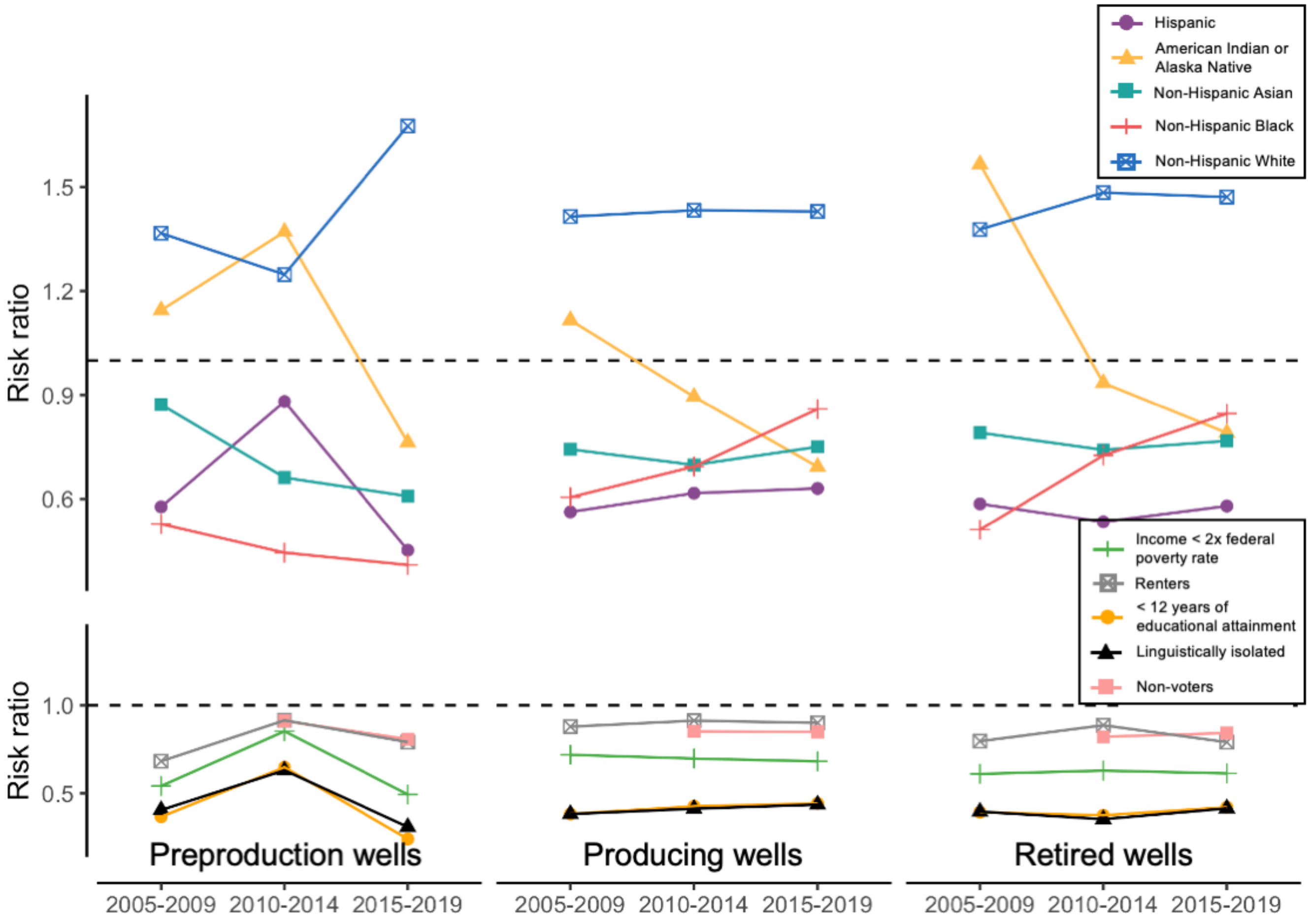

Figure S9. Estimated risk ratios for each racial/ethnic (top row) and socioeconomic group of interest (bottom). We estimated the proportion of each group in <u>Orange County</u> with any exposure to wells in one of three stages: new (left column), active (center), and retired (right). For each group and well stage, we estimated risk ratios in the three time periods included in the study: 2005 to 2009, 2010 to 2014, and 2015 to 2019. A risk ratio > 1 means the group has disproportionately high exposure compared to the Orange County representation of the group and a risk ratio < 1 means the group has disproportionately low exposure, compared to the group's representation in Orange County. Compare to Figure 3.

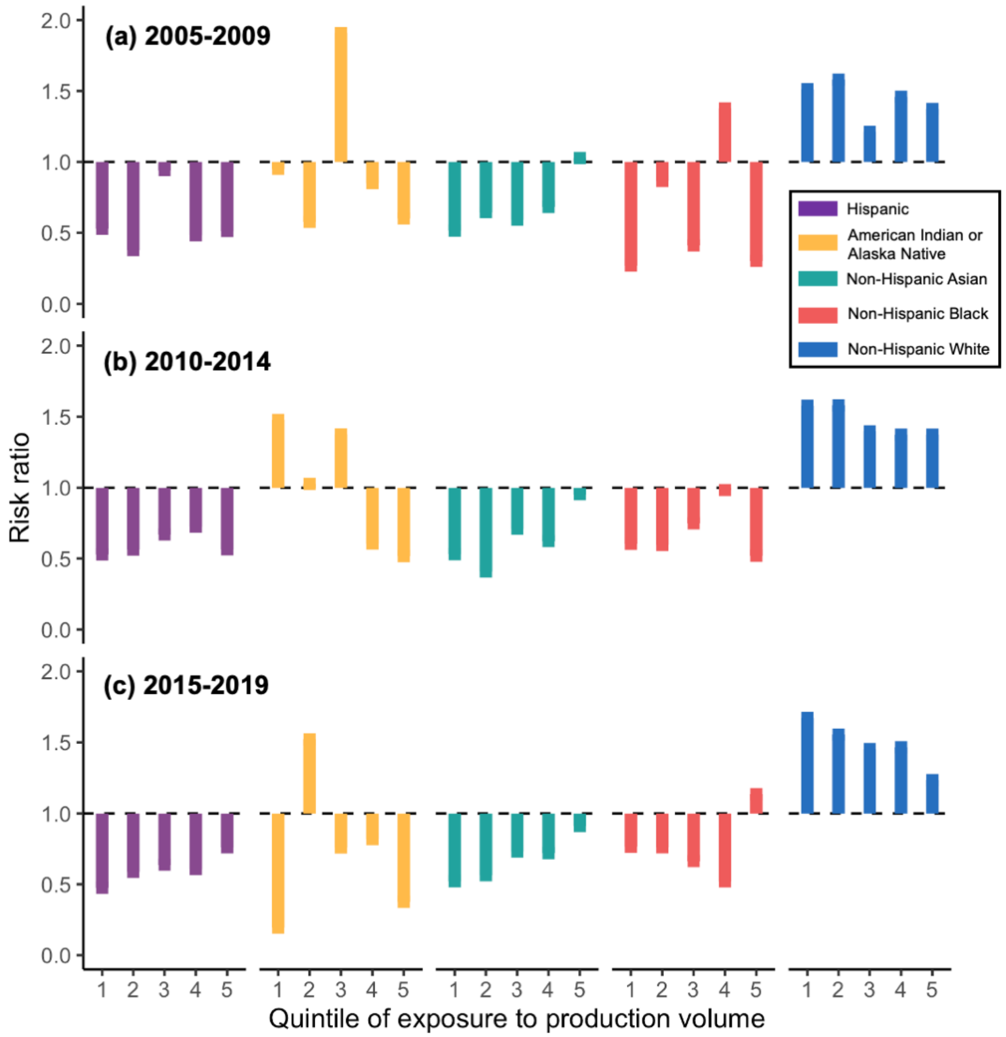

Figure S10. Among block groups in <u>Orange County</u> within 1 km of active wells, we estimated risk ratios for exposure to cumulative oil and gas production volume stratified by quintile of exposure (from least exposure, 1, to highest exposure, 5). For each racial/ethnic group, we estimated risk ratios by comparing the proportion of the group that was exposed relative to the proportion of the group within the county. A risk ratio > 1 means the group has disproportionately high exposure compared to the county-wide proportion and a risk ratio < 1 means the group has disproportionately low exposure. Compare to Figure 4.

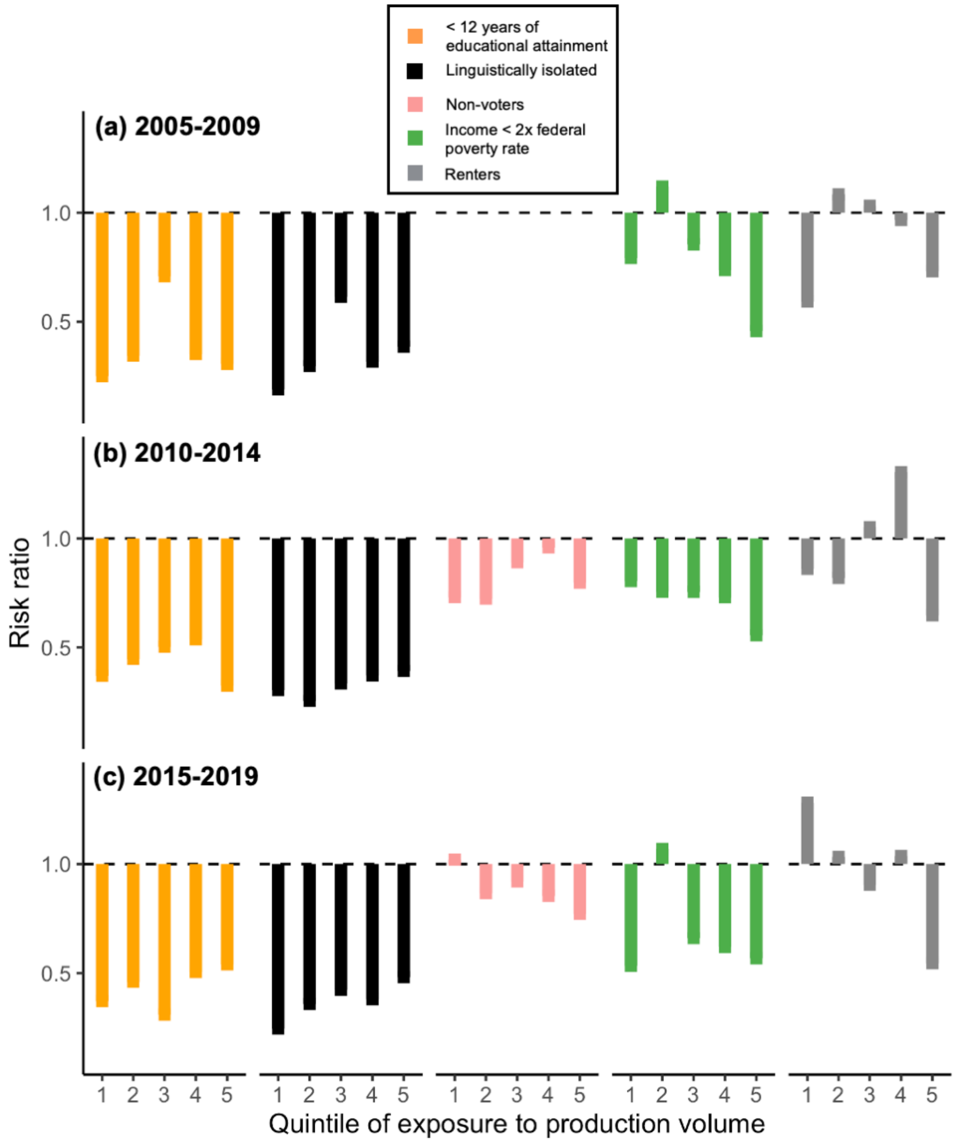

Figure S11. Among block groups in Orange County within 1 km of active wells, we estimated risk ratios for exposure to cumulative oil and gas production volume stratified by quintile of exposure (from least exposure, 1, to highest exposure, 5). For each group identified by socioeconomic indicators, we estimated risk ratios by comparing the proportion of the group that was exposed relative to the proportion of the group within the county. A risk ratio > 1 means the group has disproportionately high exposure compared to the county-wide proportion and a risk ratio < 1 means the group has disproportionately low exposure. Compare to Figure S1.

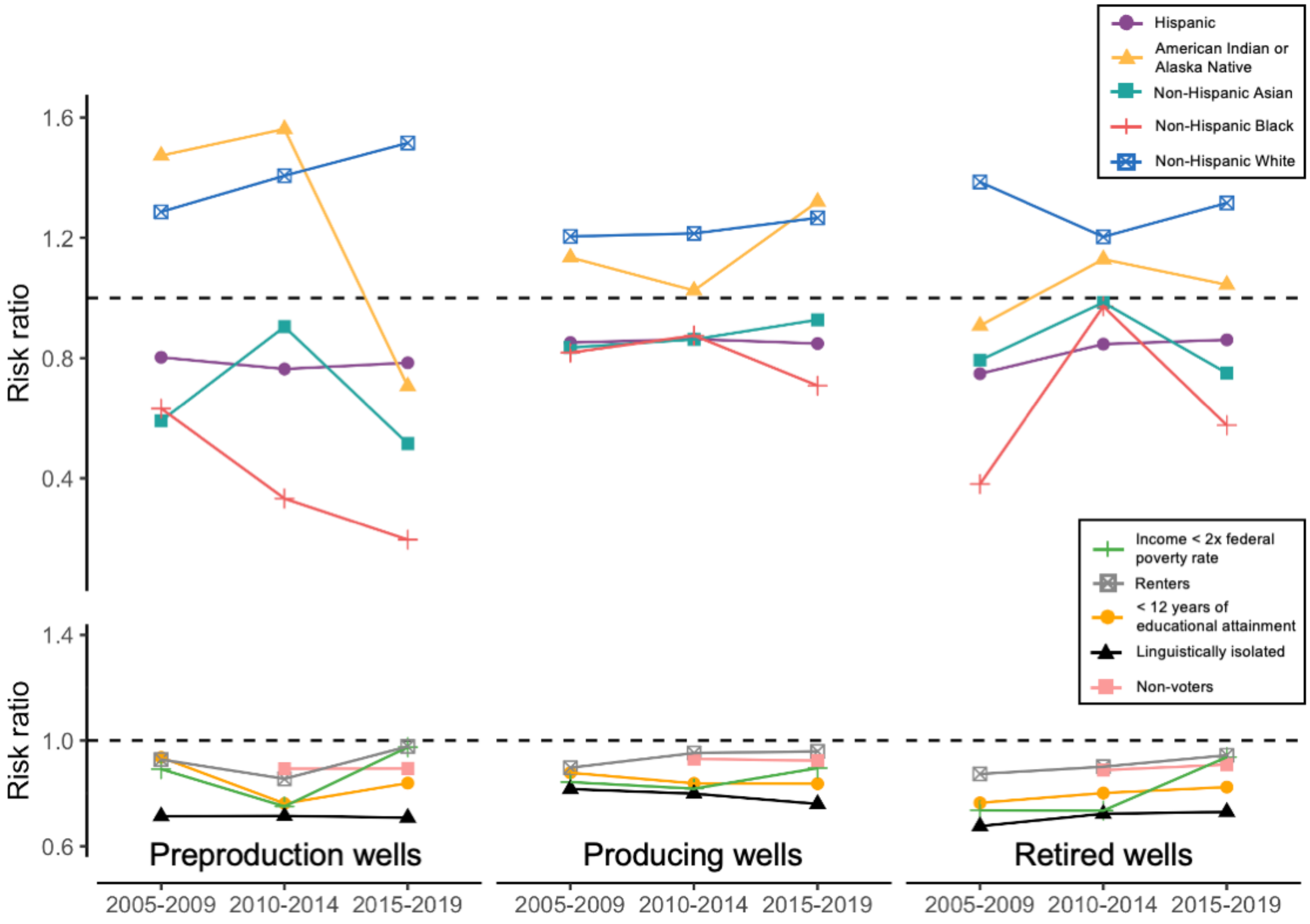

Figure S12. Estimated risk ratios for each racial/ethnic (top row) and socioeconomic group of interest (bottom). We estimated the proportion of each group in Kern County with any exposure to wells in one of three stages: new (left column), active (center), and retired (right). For each group and well stage, we estimated risk ratios in the three time periods included in the study: 2005 to 2009, 2010 to 2014, and 2015 to 2019. A risk ratio > 1 means the group has disproportionately high exposure compared to the Kern County representation of the group and a risk ratio < 1 means the group has disproportionately low exposure, compared to the group's representation in Kern County. Compare to Figure 3.

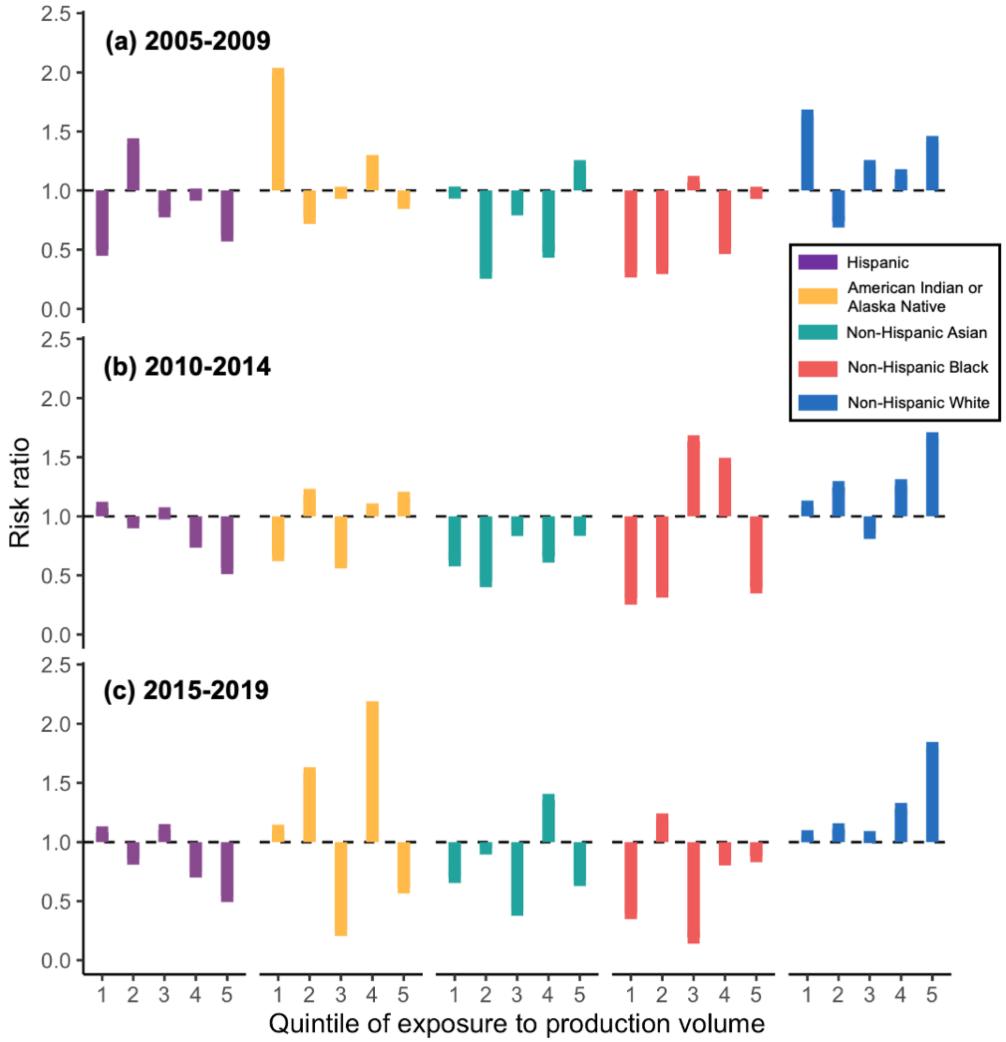

Figure S13. Among block groups in Kern County within 1 km of active wells, we estimated risk ratios for exposure to cumulative oil and gas production volume stratified by quintile of exposure (from least exposure, 1, to highest exposure, 5). For each racial/ethnic group, we estimated risk ratios by comparing the proportion of the group that was exposed relative to the proportion of the group within the county. A risk ratio > 1 means the group has disproportionately high exposure compared to the county-wide proportion and a risk ratio < 1 means the group has disproportionately low exposure. Compare to Figure 4.

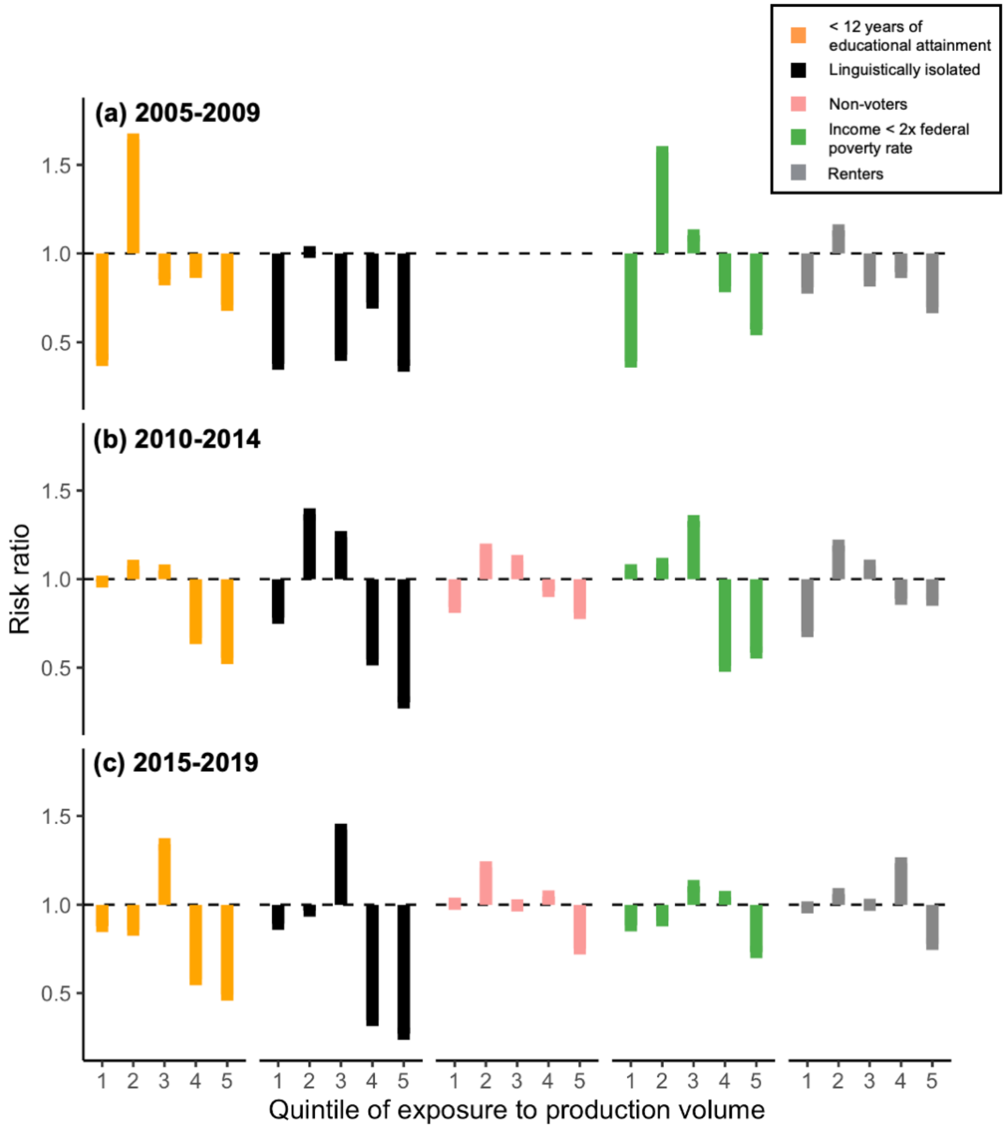

Figure S14. Among block groups in Kern County within 1 km of active wells, we estimated risk ratios for exposure to cumulative oil and gas production volume stratified by quintile of exposure (from least exposure, 1, to highest exposure, 5). For each group identified by socioeconomic indicators, we estimated risk ratios by comparing the proportion of the group that was exposed relative to the proportion of the group within the county. A risk ratio > 1 means the group has disproportionately high exposure compared to the county-wide proportion and a risk ratio < 1 means the group has disproportionately low exposure. Compare to Figure S1.
